# Dexmedetomidine-mediated sleep phase modulation ameliorates motor and cognitive performance in a chronic blast-injured mouse model

**DOI:** 10.3389/fneur.2022.1040975

**Published:** 2022-11-01

**Authors:** Yelena Bibineyshvili, Nicholas D. Schiff, Diany P. Calderon

**Affiliations:** ^1^Department of Anesthesiology, Weill Cornell Medical College, New York, NY, United States; ^2^Feil Family Brain and Mind Research Institute, Weill Cornell Medical College, New York, NY, United States

**Keywords:** sleep/wake cycle disturbance, blast injury model, dexmedetomidine, motor behavior, cognitive behavior, chronic blast injury

## Abstract

Multiple studies have shown that blast injury is followed by sleep disruption linked to functional sequelae. It is well established that improving sleep ameliorates such functional deficits. However, little is known about longitudinal brain activity changes after blast injury. In addition, the effects of directly modulating the sleep/wake cycle on learning task performance after blast injury remain unclear. We hypothesized that modulation of the sleep phase cycle in our injured mice would improve post-injury task performance. Here, we have demonstrated that excessive sleep electroencephalographic (EEG) patterns are accompanied by prominent motor and cognitive impairment during acute stage after secondary blast injury (SBI) in a mouse model. Over time we observed a transition to more moderate and prolonged sleep/wake cycle disturbances, including changes in theta and alpha power. However, persistent disruptions of the non-rapid eye movement (NREM) spindle amplitude and intra-spindle frequency were associated with lasting motor and cognitive deficits. We, therefore, modulated the sleep phase of injured mice using subcutaneous (SC) dexmedetomidine (Dex), a common, clinically used sedative. Dex acutely improved intra-spindle frequency, theta and alpha power, and motor task execution in chronically injured mice. Moreover, dexmedetomidine ameliorated cognitive deficits a week after injection. Our results suggest that SC Dex might potentially improve impaired motor and cognitive behavior during daily tasks in patients that are chronically impaired by blast-induced injuries.

## Introduction

Anatomical and functional damage to the brain after physical trauma due to an explosive blast produces acute and chronic outcomes affecting cognition, emotion, and motor behavior (1–3). Sleep/wake disturbances frequently contribute to these sequelae (4, 5). For instance, 30–70% of individuals with mild, moderate, or severe traumatic brain injury report sleep-wake cycle disturbances up to 3 years after injury, contributing to memory, cognitive and psychological distress (4). Specifically, 80% of blast-injured patients reported poor sleep quality and 54% reported sleep fragmentation (6). Importantly, < 20% of patients indicated that symptoms had improved after years of injury. Although acute and chronic sequelae both have significant impact on patients, current therapeutic approaches are focused on acute post-injury stages rather than long-term treatments. Moreover, electrophysiological sleep/wake cycle hallmarks such as changes in the power of single electroencephalographic (EEG) frequency bands, coherence, and cross-frequency coupling that predict recovery outcome remain controversial (4) since they depend on the extent of the damage, type of injury and neurological findings (7). However, robust EEG indices would aid assessment of brain trauma severity (8, 9), recovery (7), and treatment outcome. For example, such cortical biomarkers may be associated with behaviors that objectively quantify recovery of levels of arousal (10) and reestablishment of the sleep/wake cycle.

It has also been hypothesized that electrophysiologic signals related to sleep/wake cycle are associated with plasticity changes. For instance, spindles, which are corticothalamic transient oscillatory events (11), are associated with increased responsiveness to synaptic stimuli and formation of new dendritic spines resulting in permanent structural plasticity changes (12). Indeed, multiple studies have linked sleep spindles to learning and memory (13, 14). In particular, non-rapid eye movements (NREMs) promote declarative and procedural learning, and improve performance in motor learning tasks in rodents and humans (12, 15). We propose that pharmacologic modulation of NREM spindles and EEG power (amount of activity in certain frequency bands of the EEG) can improve the performance of chronically injured mice in various tasks.

Dexmedetomidine (Dex) is a selective alpha-2 adrenoceptor agonist that is used in post-operative patients as a sedative and analgesic and has minimal respiratory effects. Dex mimics normal physiologic sleep and preserves sleep spindle features suggesting that spindle-generated neuronal mechanisms may reflect physiological NREM (stage 2) (16). Moreover, Dex increases spindle activity in rodents (17). Therefore, we hypothesized that administering Dex to chronically injured mice during the sleep phase can improve motor and cognitive tasks.

We previously generated a mouse model of secondary blast injury (SBI) in the brain (18) using local high-pressure waves that propel shrapnel, and studied cognitive, motor and mood deficits. SBIs are injuries caused by fragments and debris propelled by high-pressure waves during explosions (19). Using this model, we found that female and male mice showed significant changes in motor behavior linked to degeneration of the cerebellum (Crus 1), ventral tegmental area, sub-thalamic nucleus and zona incerta that likely alter the basal ganglia function. However, cognitive impairment associated with degeneration in hippocampus, subiculum and the retrosplenial area was prominent in male, but not female, mice. In this study, we used male SBI mice to test our hypothesis using a battery of behavioral tests.

Here we map the natural evolution of EEG activity and behavior after SBI and the effect of subcutaneous Dex on multiple motor and cognitive tasks executed by chronically impaired mice.

## Materials and methods

**Figure 1:**
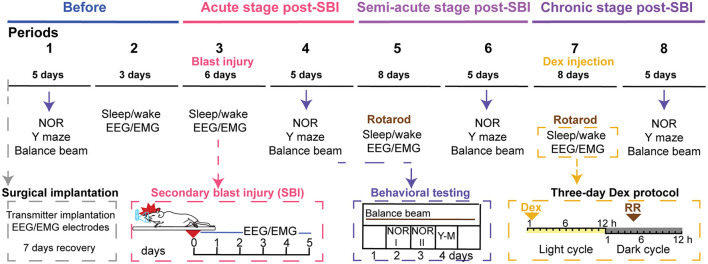
Timeline of the longitudinal analysis of the sleep/wake cycle and behavior in SBI and control mice. The schematic illustrates the timeline of the repeated tests applied to blast injured and control mice before and several weeks after blast injury. The top line shows the time scale divided into periods. Seven days before period one, animals were implanted with a wireless transmitter (gray box). Then in period 1 (5 days), mice were exposed to a battery of motor and cognitive tests, including novel object recognition (NOR), Y maze(Y-M), and balance beam. After this evaluation, the sleep/wake cycle was monitored for 24 h on three consecutive days (period 2). Next, on day 0 of period 3, mice were subjected to a blast and sham procedure, respectively, while animals were anesthetized. We monitored EEG and EMG activity immediately after SBI and for six consecutive days. Later in period 4, we repeated motor and cognitive testing (blue box). After that, we studied EEG/EMG activity in period 5 for 8 days, and mice executed the rotarod (RR). We repeated the cognitive and motor tasks for the third time and for 5 days (period 6). Subsequently, while we were monitoring EEG/EMG activity (period 7), we injected Dex (0.15 mg/kg) within the first hour of the light cycle, and 16 h later, mice ran on the rotarod (orange box). Lastly, we evaluated cognitive and motor behavior a week after Dex injection (period 8).

All use of laboratory animals was consistent with the *Guide for the Care and Use of Laboratory Animals* and approved by the Weill Cornell IACUC (Protocol No. 2016-0054). Ten to twelve week old C57BL/6 wildtype male mice (Jackson labs) were maintained on a reverse cycle (lights turned off at 9:00 h and on turned on at 21:00 h), with food and water provided *ad libitum*. We used a total of 67 animals. The SBI group was initially comprised of 45 animals. We assigned mice to the SBI and sham group using the *rand* function (Matlab) that extracts the ID number of a mouse from an array without replacement. Within the SBI group, five mice died immediately following SBI, and ten mice were euthanized due to significant distress during recovery (IACUC guidelines). We thus examined motor and cognitive behavior in 30 SBI mice and 22 sham mice before and after the blast injury. In addition, we examined EEG activity before and after injury in 17 SBI and 11 sham mice (Figure 1). We examined Dex/vehicle modulation of the sleep/wake cycle in all mice.

### EEG transmitter implantation

Mice included in the study were weighed and anesthetized in an induction chamber using an initial isoflurane concentration of 4% by volume in O_2_. The eyes were protected with ophthalmic ointment. Animals were then transferred to a stereotaxic frame, and anesthetic concentration was maintained using a nose cone at a 1.25% (~1MAC). Temperature was maintained at ~37°C using a temperature regulator coupled to a rectal temperature probe (CWE Inc). The skull was fixed to the stereotaxic frame, animal hair was removed, and the surgical area cleaned using alternating betadine and ethanol sterile pads. The wound edge was infiltrated with local anesthetic (bupivacaine 0.5%). After opening an incision in the scalp, we created a subcutaneous pocket along the animal's dorsal flank and placed the body of the transmitter (model HD-X02; DSI) into the pocket, ensuring biopotential leads were oriented cranially. The first lead was placed in a craniotomy made at stereotaxic coordinates targeting the cingulate cortex (AP: 1.5 mm; ML: −1 mm from bregma). The reference lead was placed within the posterior parietal area. We secured the leads using dental acrylic. Then, the second lead was placed through the trapezoid muscle for electromyography (EMG) perpendicular to the long axis of muscle fiber bundles and fixed with a suture. We provided analgesic (Flunixin 2.5 mg/Kg) and saline postoperatively. We monitored weight and temperature over the course of 7 days, as previously reported (18). Animals were placed alone in cages to facilitate recovery and EEG/EMG monitoring.

### Secondary blast injury

We previously described an SBI mouse model (18) in which we modeled injuries caused by flying debris generated by an explosion (20). Briefly, an air blast was generated using a 6-gallon portable electric air compressor attached to a nail gun (no nails were present). The air blast triggered by the device propelled a piston (shrapnel) toward an ~2 × 2 mm area of the brain that included the frontal/prefrontal cortical-pallidal thalamocortical loops. The blast also resulted in degeneration in distant areas, including the midbrain and brainstem regions (18). Animals were induced with isoflurane (4% vol) and maintained with a (1.50% vol). Before the impact, we applied ophthalmic ointment and Flunixin 2.5 mg/Kg. The head and half of the body was inserted into a plastic tube covered with molded foam. This tube was placed perpendicular to the SBI device toward the right parietal region of the mouse. A micro-switch sensor controlled by a motor stepper held the head in position and the temperature of each animal was monitored and maintained at 36 ± 05°C using a CWE temperature controller. We provided an air blast pressure of 50 psi as previously described (18). Sham mice underwent a similar procedure except that the blast occurred several centimeters away from the head and the piston was deflected by an object placed in the apparatus. Animals were transferred straight to the home cage for EEG/EMG monitoring. A space gel pad maintained warmth during recovery.

After the induced-blast injury, animals were frequently monitored (three to four times a day) for a week. We tracked body weight, temperature, urination, and defecation. Moreover, although we provided moist food pellets in a plastic cup at the bottom of the cage, we manually fed individuals with a gavage syringe when necessary. Signs of distress and dehydration were strictly monitored, and humane treatment was provided following IACUC guidelines.

### Monitoring sleep/wake cycle

The sleep/wake cycle was assessed before and after SBI treatment by monitoring EEG/EMG activity for 24 h over several days (Figure 1; periods 2 and 3). We also examined EEG/EMG activity during the third week post-injury (period 5) to establish an intermediate point on the recovery trajectory of the sleep/wake cycle. During the fifth week after injury, we recorded EEG/EMG activity and injected Dex (period 7) to determine the effects of modulating the sleep/wake cycle in SBI mice. A receiver located under the home cage of each animal transmitted data from the implanted telemetry device to the Ponemah V 6.5 software from Data Sciences International (DSI). This system wirelessly recorded EEG, EMG activity, activity counts, and temperature while animals freely moved in their home cages. We assessed subjects throughout the dark (9:00–21:00 h) and light cycles (21:00–9:00 h) while performing telemetry recordings. No epileptiform discharges were observed in mice that survived after trauma. EEG and EMG activity were sampled at a rate of 500 Hz. Temperature and activity counts were sampled at 10 and 1 Hz respectively. All recordings were performed in a cubicle shielded from background disturbances.

### Detection of sleep/wake cycle stages

We extracted data from the Ponemah software (DSI) and converted it to ASCII format. We imported data to Matlab (Mathworks). Data was filtered from noise artifacts and the delta (2–4 Hz), theta (5–8 Hz), alpha (8–13 Hz), beta (16–31 Hz), and gamma (32–100 Hz) power were calculated using the Thomson multitaper method implemented in the Chronux toolbox in Matlab (21, 22). To compute cortical spectrograms, we used the function *mtspecgramc* with the following parameters*: frequency band* = *[0, 150 Hz], tapers* = *[3, 5], movingwin* = *[5, 0.1] s*. For the EMG, we applied the following parameters*: frequency band [5, 170 Hz] and tapers [3, 5]. We* then classified states as NREM, REM, awake, or undetectable, based on 5 s traces.

#### Detection of awake state

An active awake state is characterized by high muscle power, high gamma, and low delta power (23–25). Therefore, we defined 5-s intervals as “awake” using thresholds in EEG and EMG signals that were manually defined based on the standard deviation from the median of 1 day. To properly define the influence of cortical state levels in the established threshold, we calculated the standard deviation from the median EEG/EMG record over 1 h and compared this to the standard deviation from the median of several EEG/EMG recordings taken over 24 h and several days. In order to establish the threshold, we then calculated the gamma/delta ratio and estimated that this ratio should be 0.5 times above the standard deviation from the median gamma/delta ratio calculated within a 1-h record. After Matlab semi-automatic detection, results were visually inspected to confirm proper detection.

#### Detection of NREM

The NREM phase is characterized by high delta and low muscle power (25, 26). Therefore, we determined “NREM” 5-s intervals on traces not previously defined as awake using a threshold of a delta/EMG power ratio exceeding one standard deviation calculated over 1 h record using Matlab. The detection results were visually inspected to confirm proper NREM identification.

#### Detection of REM

We defined the REM stage as the ratio between theta/(delta × muscle power) (23). Thus, we determined “REM” in 5-s intervals on traces not previously defined as awake or NREM using a manual threshold (lower limit) established by the theta/(delta × muscle power) ratio standard deviation calculated over the course of 1 h. To properly define the threshold according to the behavioral states of the subject, we calculated the standard deviation from the median from an hour-long EMG record and compared it to the standard deviation from the median EMG recorded over several days.

#### Detection of spindles

Sleep spindles are transient rhythmic oscillatory EEG events usually ranging between 11 and 16 Hz (11). These events are a physiological hallmark of NREM sleep in humans (27). Since spindle parameters vary with age and pathological condition (28, 29), we band passed EEG traces using a range of 10–20 Hz. Then, we normalized the power of the filtered signal and selected power peaks exceeding two absolute EEG standard deviations from the mean. We defined a *single spindle* from EEG traces containing more than five selected power peaks, an interval exceeding 0.2 s in length, and an inter-peak interval < 0.16 s. We visually inspected spindle detection using Matlab by applying the *mtspecgramc* function from Chronux and the following parameters for EEG: frequency band = [0, 40 Hz], tapers = [3, 5], movingwin = [1, 0.1] s.

We estimated several parameters for every spindle in the NREM stage, including amplitude, intra-spindle frequency, duration, symmetry, cycle number, and density. Therefore, *spindle amplitude* was defined as the difference between maximal positive and minimal negative power peaks (28). *Cycle number* was defined as the number of positive power peaks. The intra-spindle frequency was defined as the cycle number/duration of the spindle, and symmetry was defined as a position of maximal positive power peak relative to spindle duration (28). Lastly, *sleep spindle density* was defined as the count of detected spindles divided by the NREM duration.

### Behavioral testing

Before any behavioral assessment, we acclimatized mice for 30 min to the respective testing room. All behavioral testing occurred in the dark part of the light/dark cycle.

#### Balance beam

We placed mice on a wooden beam elevated 36 cm from the ground. The length of the beam was 54.5 cm, and its diameter was 1.2 cm. A dark safe box was placed at one end (LUT = 8). While in training, mice were gently placed for 30 s in the dark box. In contrast, mice were placed on the beam on the opposite side of the safe box during the trials. This side was illuminated with a lamp (LUT ≥ 1000). For this experiment, we performed three trials on five consecutive days (Figure 1; periods 4, 6, and 8th) and cleaned the beam and box with Clidox-S between trials. We recorded trials using Ethovision (Noldus) and used the software to divide the beam into five segments (10 cm each). Subsequently, we determined the average velocity reached in crossed segments.

#### Accelerating rotarod test

We used the rotarod (Rotamex-5, Columbus Instruments) as previously described (18) to assess motor performance during post-SBI recovery. Briefly, we applied a paradigm in which the rod was accelerated at 0.1 cm/s throughout the trial. The speed increased from 0 to 40 cm/s over 400 s. The rotarod test was given on days where EEG/EMG was also recorded (Figure 1; periods 5 and 7). We completed each EEG/EMG recording at noon. Then, we ran the rotarod test for an hour and returned the animals to the receivers to start a new EEG/EMG recording. We cleaned the rod with Clidox-S between trials. To measure the motor performance specifically, we trained mice before Dex injection (Figure 1; period 5) for several days until mice reached a motor learning plateau. Then, we executed the following rotarod assessment protocol (Figure 1; period 7): (1) 3 days before Dex to secure a steady plateau; (2) during the days of Dex injection, and (3) after Dex treatment. We subjected mice to 5 trials per day with a resting time of at least a minute in between trials. The Rotamex laser system automatically detected the length of time until animals lost coordination (run time).

#### Novel object recognition

Animals were habituated to a square testing chamber (40 × 40 cm) for 10 min (without any objects). During training (day 1), mice were exposed to two equidistant identical objects (object #1 and #2) in the same chamber and allowed to explore freely for 10 min. Then, 24 h after completion of training (day 2), each mouse was tested using one object previously presented object (object #2) and one unfamiliar object (object #3). The behavior was video recorded using Ethovision (Noldus). We cleaned the arena with Clidox-S after each trial.

#### Y maze

The maze comprises three symmetrical arms (spaced 120° apart) forming a Y. After room acclimation, mice freely explored the maze containing a “familiar” arm signaled with a visual cue. In addition, the maze had an arm defined as “new,” which was blocked, and a “start” arm, in which all mice were given 5-min of initial exposure to the maze. An hour later, animals were placed back for a second trial, leaving the “new” arm open and letting mice explore the maze for 2 min. We recorded mouse behavior using Ethovision (Noldus). Y maze was cleaned with Clidox-S after each trial.

### Dexmedetomidine injection

Animals were randomly injected with Dex (0.1 mg/Kg) (30) or saline (vehicle) during period 7 for 3 consecutive days. We treated mice with Dex for 3 days as others found that this time frame was sufficient to reverse cognitive dysfunction in a mouse inflammation model (31). In addition, a 3-day treatment allowed us to obtain EEG activity before, during, and after treatment without altering our protocol of EEG/EMG and behavior examination. Injections were administered at 21:00 h when the light cycle started. Animals were immediately returned to their cages to monitor EEG/EMG activity. Animals performed the rotarod task the following day at around 13:00 h.

Once EEG/EMG monitoring and behavioral testing were completed, we terminally anesthetized animals and intracardially perfused them with paraformaldehyde (4%), followed by confirmation of electrode implantation in the correct area of the brain.

### Statistical analyses for experiments

We performed a total of five different replicates between December 2020 and April 2022.

We excluded two animals from the study because they showed signs of significant distress after transmitter implantation. We also extracted and kept the subset of useable data from two animals that experienced lead detachment and, therefore, noisy EEG/EMG signals post-SBI. All trials collected through Ethovision were visually inspected to assure acquisition and detection accuracy. All data except rotarod and intra-spindle frequency were normalized to task performance and EEG activity before blast injury (Figure 1; period 1). Given the results of the Jarque-Bera test, we defined our data as non-parametric. Therefore, we applied the Mann–Whitney *U* test for two-group comparison and one-way Kruskal–Wallis test followed by Dunn's post hoc test with Bonferroni correction for 3 groups (sham, SBI-HD and SBI-LD subgroups). Lastly, we applied Friedman test and Wilcoxon rank test with Bonferroni correction for comparison of same group on different days. The analytic code written in Matlab or R Studio used to conduct the detection and analysis of NREM, REM, and awake states as well as spindles through 24 h is available from the corresponding author and can be accessed *via* GitHub.

#### Novel object recognition (NOR)

We excluded 14 out of 196 trials due to environmental interference and three mice because of similarity issues between “familiar” and “novel” objects during the pilot study. In addition, we excluded trials in which the subject spent < 10 s exploring the object because it is difficult to confirm whether the animal was able to explore/discriminate (32). Frequency and total time of visits to each object was analyzed using the open source program OptiMouse (33) written in Matlab. This software accurately determined the animal's nose position in zones of interest (10 × 10 cm) surrounding a given object and calculated the time spent by the animals exploring object#1 (time1) and object#2 (time2) on day 1 and object#3 (time3) and object#2 (time4) on day 2. We established the new object preference as $\left(\;\frac{time3}{time4\;}\;\right)$/($\frac{time1}{time2}$) and corrected for possible unequal preference at day 1. We compared NOR execution at periods 4, 6, and 8 (a week after Dex injection; Figure 1).

#### Y maze

We excluded six trials out of 208 because of poor camera detection (the camera lost subject detection for more than 10% of the trial). We also excluded trials where the animal spent < 15 s in the “new” or “familiar” arm. Lastly, if the total number of visits to all arms was < 7 during the trial, this trial was excluded. We calculated preference for the “new” arm as the time spent in “new” arm vs. “familiar” arm ratio (34). Preference for a new arm was calculated for periods 4, 6, and 8 (a week after Dex injection; Figure 1).

#### Rotarod

We excluded 24 out of 3,570 trials due to environmental interference. In addition, we excluded a single animal as test conditions were not equivalent to other trials in the rotarod. We compared time on rotarod during periods 5 and 7 (Figure 1).

#### Balance beam

We excluded 8 out of 208 trials due to environmental interference or unsuccessful learning. The velocity on the beam was averaged for all trials per day and through 5 days at periods 1, 4, 6 and 8th respectively.

## Results

### Secondary blast injury alters the sleep wake cycle

**Figure 2:**
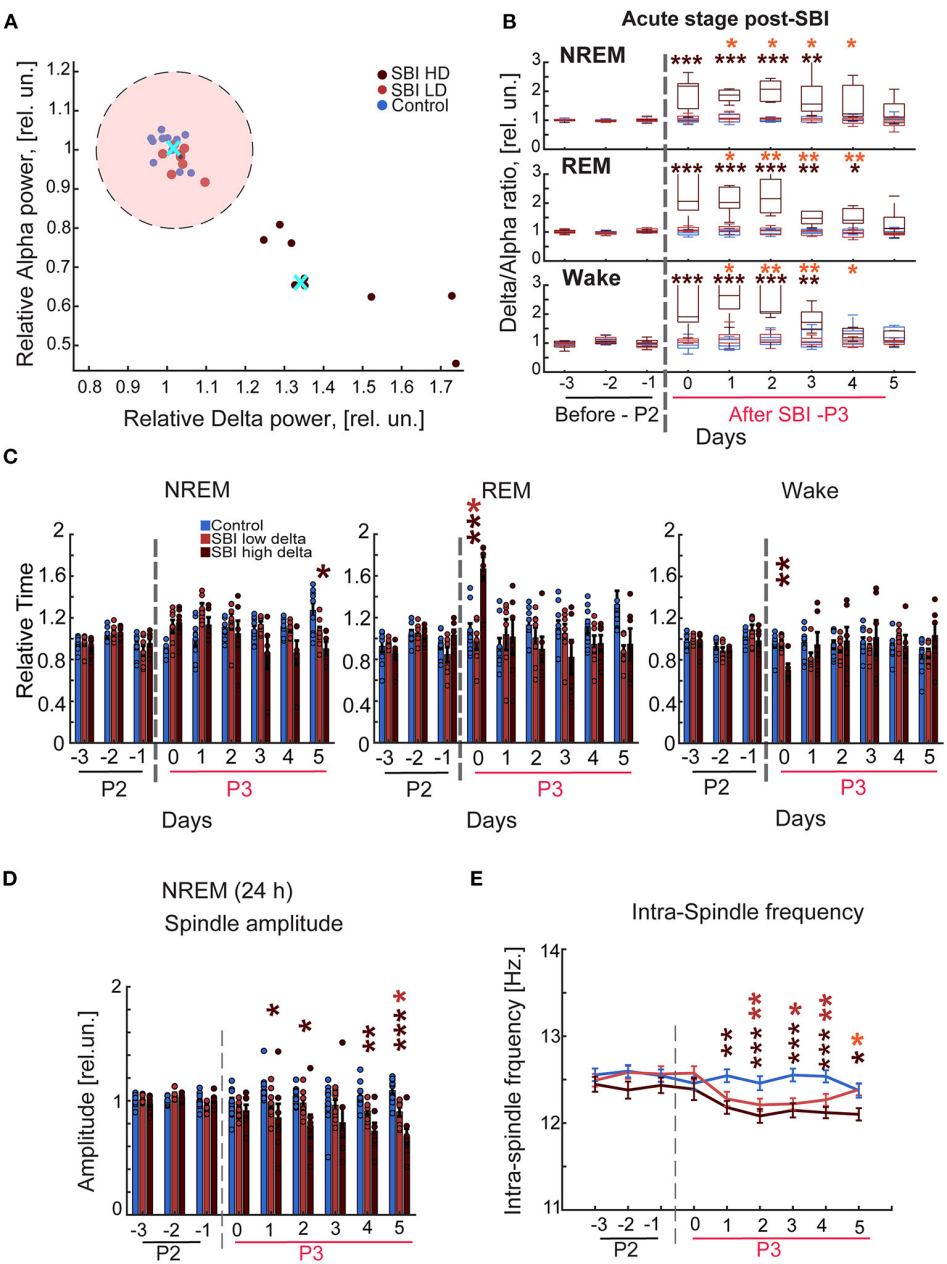
Blast injured mice exhibit an excessive sleep pattern during the acute stage post-injury. **(A)** Representative scatter plot of the delta-alpha scores obtained from SBI and sham mice during day 2 after trauma (period 3, P3) and normalized to the delta/alpha ratio before blast injury (period 2; P2). Data were clustered into two groups using the *K*-means function: mice with a high (HD) and low (LD) delta/alpha ratio. The cluster centroids (cyan cross) represent the average of delta and alpha power relative scores, and the pink circle depicts 90% of the mean distance between the centroid of the cluster containing control animals and LD. Carmine dots represent SBI mice assigned to the cluster of low delta/alpha ratio (*n* = 8); brown dots, SBI mice assigned high ratio (*n* = 8), and blue dots correspond to the control group (*n* = 11). **(B)** Delta alpha ratio differences between SBI-HD (*n* = 7), SBI-LD (*n* = 8), and sham mice (*n* = 11) in NREM, REM, and Wake state before and after blast induced-injury (dashed gray line). **(C)** NREM, REM, and awake episode duration over 24 h in SBI-HD (*n* = 7), SBI-LD (*n* = 8) and sham mice (*n* = 11) and normalized to averaged episode duration before SBI. **(D)** Average peak domain amplitude (mean ± S.E.M.) in microvolts of the detected spindles in 24-h periods and normalized to the averaged peak found before SBI represented in relative units (rel.un.). **(E)** Averaged intra-spindle frequency distribution (mean ± S.E.M.) in detected spindles during 24 h in SBI-HD, SBI-LD and control mice days before and after causing SBI. We employed Kruskal Wallis and Dunn's test with Bonferroni correction, *p < 0.05, **p < 0.01, ***p < 0.001. Carmine asterisks represent differences between sham and SBI-LD, brown asterisks depict differences between SBI-HD and sham mice, and the orange asterisk display differences between SBI-HD and SBI-LD groups.

Individuals with mild, moderate, or severe traumatic brain injury report persistent sleep disruption (6) that contributes to cognitive and psychological distress (4). Thus, we hypothesized that SBI animal models might also have lasting sleep wake cycle disturbances. We therefore wirelessly recorded EEG/EMG activity for three consecutive days before (period 2: Figure 1) and for 5 days after blast injury (period 3: Figure 1). Our results showed sleep disturbance in the animals: we observed a significant increase in delta power during awake, NREM, and REM states in SBI compared to sham-treated mice. This shift was seen immediately after injury but continued for days (Supplementary Table 1). In contrast, alpha and gamma power was reduced in all three states, and beta frequency was significantly diminished during sleep (REM + NREM; Supplementary Table 1). Moreover, total sleep time increased, largely due to prolonged NREM duration [NREM: SBI: 1.17 ± 0.04; Ctrl: 0.9 ± 0.03 relative units (rel.un.)] *P* = 0.002—Day 0 and (SBI: 1.24 ± 0.06; Ctrl: 0.99 ± 0.06 rel.un.) *P* = 0.025—Day 1 and REM (SBI: 1.34 ± 0.1; Ctrl: 1.07 ± 0.08 rel.un. *P* = 0.075—Day 0). Awake time decreased (SBI: 0.82 ± 0.04; Ctrl: 0.96 ± 0.04 rel.un. *P* = 0.03—Day 0, and SBI: 0.74 ± 0.05; Ctrl: 0.97 ± 0.05 rel.un. *P* = 0.008—Day1) in SBI compared to control mice. Given the consistent alpha and delta power changes across the different sleep/wake stages, we followed delta/alpha ratio after injury, a method previously used to track changes in brain activity after stroke (35) and recovery after neurorehabilitation (8). In addition, multiple human patient studies have demonstrated that a dominance of delta waves is associated with a higher degree of structural injury and a lower potential for recovery compared to patients with lower delta power waves (8, 9, 36). Thus, we divided the SBI mice into two groups: (1) mice with a high delta/ alpha ratio (SBI-HD) and (2) those with low delta/ alpha ratio (SBI-LD) to analyze sleep disturbance trajectories. To assign mice to these groups, we clustered the SBI population by applying *K*-means to the delta/alpha ratio data scored over REM, NREM, and awake states (Figure 2A). The SBI-HD cohort showed significant high delta/alpha power ratio compared to controls across the first 4 days [Kruskal–Wallis (KW) test NREM *H*(2) = 13.17 day 0, 15.02 day 1, 16.73 day 2, 12.77 day 3; REM *H*(2) = 14.95 day 0, 15.61 day 1, 14.96 day 2, 13.73 day 3, and awake *H*(2) = 15.55 day 0, 16.75 day 1, 15.15 day 2, 11.10 day 3, followed by multiple comparisons using Dunn's test with Bonferroni correction (MCDBC) in NREM *P* < 0.002, REM *P* < 0.004 and awake *P* < 0.009]. The second (SBI-LD) group significantly differed from the SBI-HD group (difference from SBI-HD was significant in: NREM *P* < 0.05, REM *P* < 0.05, and awake *P* < 0.05, days 1–4). SBI-LD mice showed no change in delta/alpha power ratio compared to controls (NREM *P* > 0.05, REM *P* > 0.05, and awake *P* > 0.05, days 0–4 respectively; Figures 2A,B). In addition, SBI-HD subjects showed an increased REM time (SBI-HD: 1.67 ± 0.1; Ctrl: 1.07 ± 0.08 rel.un. *P* = 0.002) and reduced awake time (SBI-HD: 0.7 ± 0.08; Ctrl: 0.96 ± 0.04 rel.un. *P* = 0.007; Figure 2C). We observed a reduction in NREM-spindle amplitude detected in 24-h periods during days 0–5 in SBI-LD mice [KW, *H*(2) = 6.29, 8.37, 11.9, 15.93 followed by MCDBC *P* < 0.05; Figure 2D], and a shift to lower frequencies within the intra-spindle oscillations (Figure 2E; Day 2: 12.2 ± 0.0, Day 3: 12.2 ± 0.0, and Day 4: 12.2 ± 0.0). KW, *H*(2) = 19.25, 18.35, 31.11 followed by MCDBC *P* < 0.05, days 2–4), but more prominently in SBI-HD mice (Day 1:12.18 ±,0.0, Day 2:12.08 ± 0.0, Day 3:12.15 ± 0.0, Day 4:12.1 ± 0.0, Day 5:12.1 ± 0.0; *P* < 0.001, and Days 1–4, *P* = 0.03, day 5) compared to the intra-spindle frequency from the control group (Day 1:12.5 ± 0.0, Day 2:12.4 ± 0.0, Day 3:12.5 ± 0.0, and Day 4: 12.5 ± 0.0). All together, these results suggest that the SBI-HD, and to lesser degree SBI-LD, mice exhibit excessive sleep during the acute post-SBI stages and additional sleep disruptions in the following days.

To examine how the functional state of the brain evolved over time post-SBI, we assessed EEG/EMG activity in mice during period 5 which corresponds to the third week post-injury (semi-acute stage). This period equates with the phase in which human patients undergo adaptive and maladaptive neural changes after traumatic brain injury (37). Our results show that the SBI group in general had a slight increase in theta power during REM (day 1: *P* = 0.004, day 2: *P* = 0.03, day 3: *P* = 0.06, and day 4: *P* = 0.04) and awake states (day 2: *P* = 0.04, day 3: *P* = 0.02, day 4: *P* = 0.05, day 5: *P* = 0.009, and day 6X: *P* = 0.07) while maintaining low alpha power in the awake state (day 1: *P* = 0.019, day 2: *P* = 0.04, day 3: *P* = 0.04, day 4: *P* = 0.02, day 5: *P* = 0.04, and day 6: *P* = 0.07) with respect to the control group. Further analysis showed that SBI-LD mice increased theta power during the awake state [KW, *H*(2) = 6.94, 5.51, 7.02, and 5.57 days 3–5, and 8; MCDBC day 3: *P* = 0.03, day 4: *P* = 0.06, day 5: *P* = 0.04, and day 8: *P* = 0.05] and the SBI-HD group kept low the alpha power during REM compared to the control and SBI-LD group [KW, *H*(2) = 7.83, 7.27, 7.48, 6.1 in days 1–3 and 8, MCDBC, SBI-HD vs. SBI-LD *P* = 0.016 Day 1, 2 and SBI-HD vs. sham day 3: *P* = 0.03, day 8: *P* = 0.04] and the awake state [KW, *H*(2) = 7.12, 9.25, 8.2, and 7.62; days 2–5], MCDBC, sham vs. SBI-HD day 2: *P* = 0.02, day 3: *P* = 0.007, day 4: *P* = 0.01, and day 5: *P* = 0.02 (Supplementary Table 2). Delta power was restored to baseline conditions in SBI-HD mice. Therefore delta/alpha power did not significantly change in period 5 in NREM [KW, *H*(2) = 0.56, 0.79, 0.40, 0.23, 0.71, 0.94, 0.57, 0.86]; REM [KW, *H*(2) = 1.11, 0.29, 1.08, 1.38, 0.91, 0.74, 0.50, 1.01; and awake states KW, *H*(2) = 0.31, 0.11, 0.14, 0.23, 0.77, 2.67, 0.99, 0.5, MCDBC, *P* > 0.05 in NREM, REM and awake, days 1–8 from Period 5] (Supplementary Figure 1A). In addition, the total sleep time resembled sham and SBI-LD groups in REM, *P* > 0.05 and NREM, *P* > 0.05 in all days from 1 to 8. Notably, the spindle amplitude tended to a lower level, KW, *H*(2) = 3.25, 9.18, 3.94, 5.36, 6.36, 3.56, 4.38, 3.02 MCDBC in NREM (SBI-HD vs. control day 2: *P* = 0.02, day 4: *P* = 0.08, and day 5: *P* = 0.04), and the intra-spindle frequency stayed significantly low [day 2:12.50 ± 0.1, 12.35 ± 0.0, 12.52 ± 0.0, 12.18 ± 0.0, 12.25 ± 0.0, 12.24 ± 0.0, 12.18 ± 0.0, 12.18 ± 0.0; KW, *H*(2) = 2.73, 17.71, 6.84, 27.34, 27.77, 18.19, 28.14, 19.88 MCDBC in NREM (SBI-HD vs. control *P* < 0.05 in days 2–8; Supplementary Figures 1C,D)].

Next, we assessed the sleep/wake cycle in period 7 which corresponds to recordings during the fifth week in post-SBI (chronic stage) when functional and histological sequelae are well established (18). During this period, the theta power through REM state remained elevated in the SBI group (day 3: SBI:1.03 ± 0.05; Ctrl: 0.95 ± 0.02 rel.un. *P* = 0.048, day 4: SBI:1.06 ± 0.05 Ctrl: 0.95 ± 0.02 rel.un. *P* = 0.01, and day 5: SBI:1.03 ± 0.05; Ctrl: 0.93 ± 0.03 rel.un. Wilcoxon test *P* = 0.03), but not in the control. However, the SBI-HD alpha power during the awake state remained low [KW, *H*(2) = 6.87, 5.56, 7.17 MCDBC SBI-HD vs. control day 1: SBI-HD: 0.9 ± 0.04; Ctrl: 1.02 ± 0.018 rel.un. *P* = 0.04, day 2: SBI-HD: 0.89 ± 0.03; Ctrl: 0.98 ± 0.03 rel.un. *P* = 0.07, and day 3: SBI-HD: 0.88 ± 0.02; Ctrl: 1.04 ± 0.03 rel.un. *P* = 0.03] as well as during REM (light cycle). [KW, *H*(2) = 8.6, 5.95, 6.34,7.45 MCDBC SBI-HD vs. control day 1: SBI-HD: 0.87 ± 0.06; Ctrl: 1.05 ± 0.02 rel.un. *P* = 0.01, day 2: SBI-HD: 0.87 ± 0.07; Ctrl: 1.06 ± 0.02 rel.un. *P* = 0.055, day 4: SBI-HD: 0.85 ± 0.07; Ctrl: 1.05 ± 0.02 rel.un. *P* = 0.037, and day 5: SBI-HD: 0.81 ± 0.06; Ctrl: 1.03 ± 0.02 rel.un. *P* = 0.02]. Delta/alpha power remained unmodified (*P* > 0.05: Supplementary Figure 2A). However, the total sleep time was significantly lower during REM in SBI-LD compared to control mice (REM, *P* < 0.05 in days 2 and 5; Supplementary Figure 2B). Similarly to period 5, the spindle-feature disruptions persisted (Supplementary Figures 2C,D): the spindle amplitude stayed low in NREM, KW, *H*(2) = 7.93, 6.83, 8.87, 7.53 MCDBC (day 1: SBI-HD: 0.82 ± 0.08; Ctrl: 1.09 ± 0.05 rel.un. *P* = 0.02, day 2: SBI-HD: 0.93 ± 0.18; Ctrl: 1.14 ± 0.05 rel.un. *P* = 0.05, day 3: SBI-HD: 0.81 ± 0.04; Ctrl: 1.08 ± 0.07 rel.un. *P* = 0.008, and day 4: SBI-HD: 0.85 ± 0.09; Ctrl: 1.16 ± 0.05 rel.un. *P* = 0.03), and the intra-spindle frequency stayed shifted toward lower frequencies in NREM [day 1:12.2 ± 0.0, day 2:12.1 ± 0.1, and day 3: 12.3 ± 0.1; KW, *H*(2) = 14.39, 6.00, 19.68, 6.28, 7.78 MCDBC (SBI-HD vs. control day 1: *P* = 0.001, day 2: *P* = 0.06, and day 3: *P* = 0.0001 and SBI-LD vs. control, day 1: *P* = 0.01, day 5: *P* = 0.03)]. Together these data demonstrate chronic EEG disturbances after SBI.

### SBI chronically affects memory tasks

**Figure 3:**
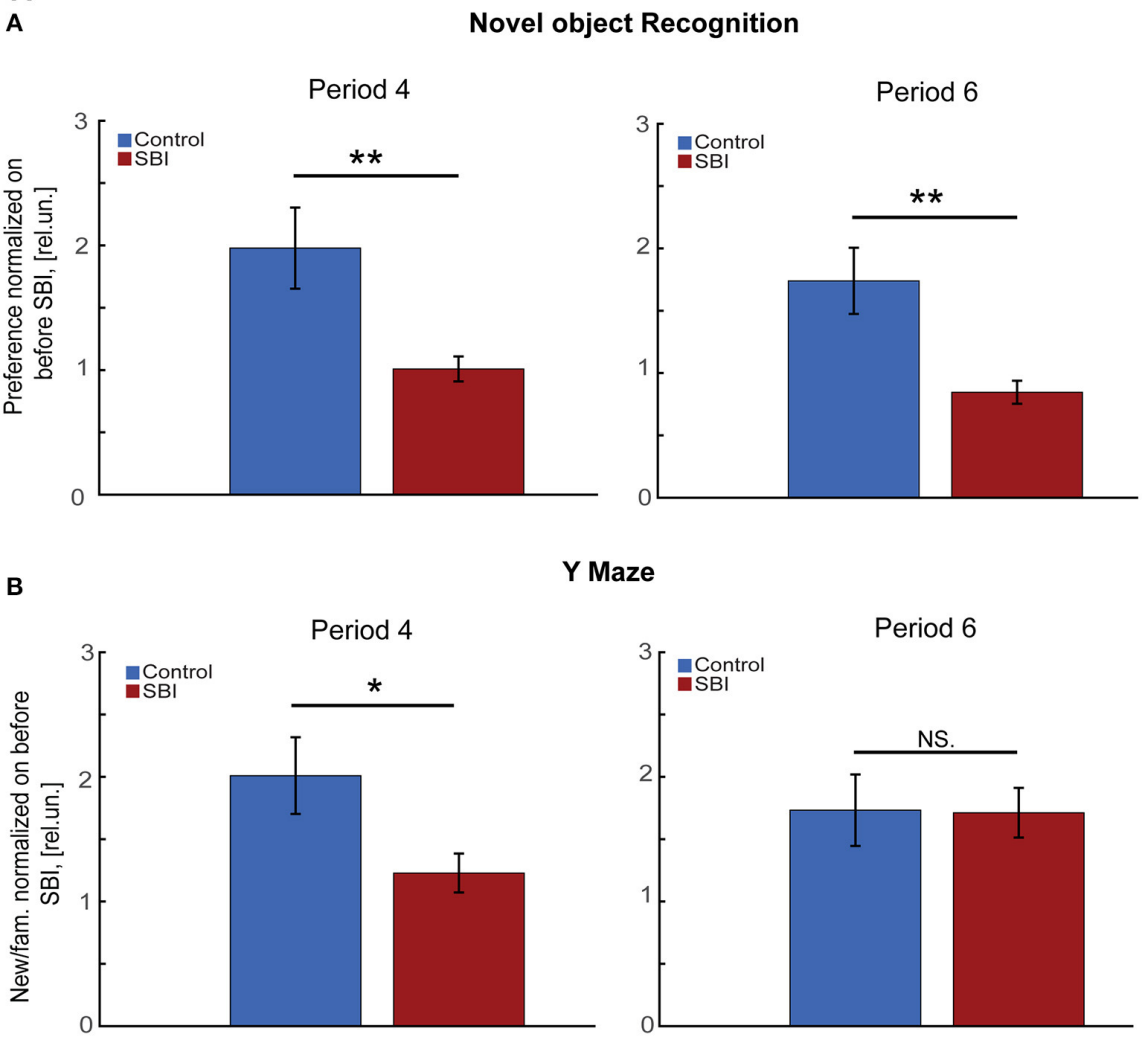
SBI chronically affects memory tasks. **(A)** Novel object preference by control (*n* = 22) and SBI mice (*n* = 30) exploring a novel object 1 (period 4) and 4 weeks (period 6) after SBI. Data were normalized to novel object preference before injury and shown in relative units (rel.un.). We applied the Mann–Whitney *U* test for a two-group comparison. **(B)** Exhibits the preference of control (*n* = 22) and SBI mice (*n* = 30) for the “new” arm of the Y Maze as the time spent in the “new” arm vs. “familiar” (fam.) arm ratio at a week and 4 weeks respectively. Data were normalized to Y Maze execution before SBI and, therefore, represented in relative units. Since the data was non-parametric, we applied the Mann–Whitney *U* test for two-group comparison *p < 0.05, **p < 0.01, and non-significant statistical differences (N.S).

Although we previously demonstrated that our blast injury model suffered cognitive impairment a week after injury using the Morris Water Maze (MWM) test, it is unknown whether this cognitive deficit persists over time and if it is associated with the described sleep disturbances. Given that mice with transmitter implantation cannot be exposed to aquatic tests, we instead subjected mice to cognitive tests such as novel object recognition (NOR), and the Y maze to assess memory consolidation (32), long and short term, and spatial memory (34). We found that through the second week after SBI (period 4 in Figure 1), SBI-treated mice showed a significantly reduced preference for the novel object compared to sham-treated controls (Figure 3A; Mann–Whitney test, SBI: 1.01 ± 0.1; Ctrl: 1.98 ± 0.33 rel.un. *P* = 0.019). Importantly, during period 6 (Figure 1; NOR through the fourth week post-SBI), this difference persisted despite changes in sleep patterns (Figure 3A; Mann–Whitney test, SBI: 0.85 ± 0.09; Ctrl: 1.74 ± 0.27 rel.un. *P* = 0.0011). We observed a similar trend when we compared the control and SBI population clustered into SBI-HD and SBI-LD groups: SBI-HD showed a significant difference compared to controls [KW, *H*(2) = 7.59, 5.05 MCDBC, *P* = 0.02 in period 4]. Likewise, when we assessed short-term memory using the Y maze (Figure 3B), we noticed a significant preference for the new arm in sham compared to SBI mice (Mann–Whitney test, SBI: 1.23 ± 0.16; Ctrl: 2.01 ± 0.31 rel.un. *P* = 0.018). However, this difference faded away at 4 weeks post-SBI (Mann–Whitney test, SBI: 1.71 ± 0.2; Ctrl: 1.73 ± 0.29 rel.un. *P* = 0.895 in period 6). In short, SBI produces long term cognitive deficits in mice and some cognitive tasks improve with changes in the sleep/wake cycle.

### SBI chronically disrupts motor tasks

**Figure 4:**
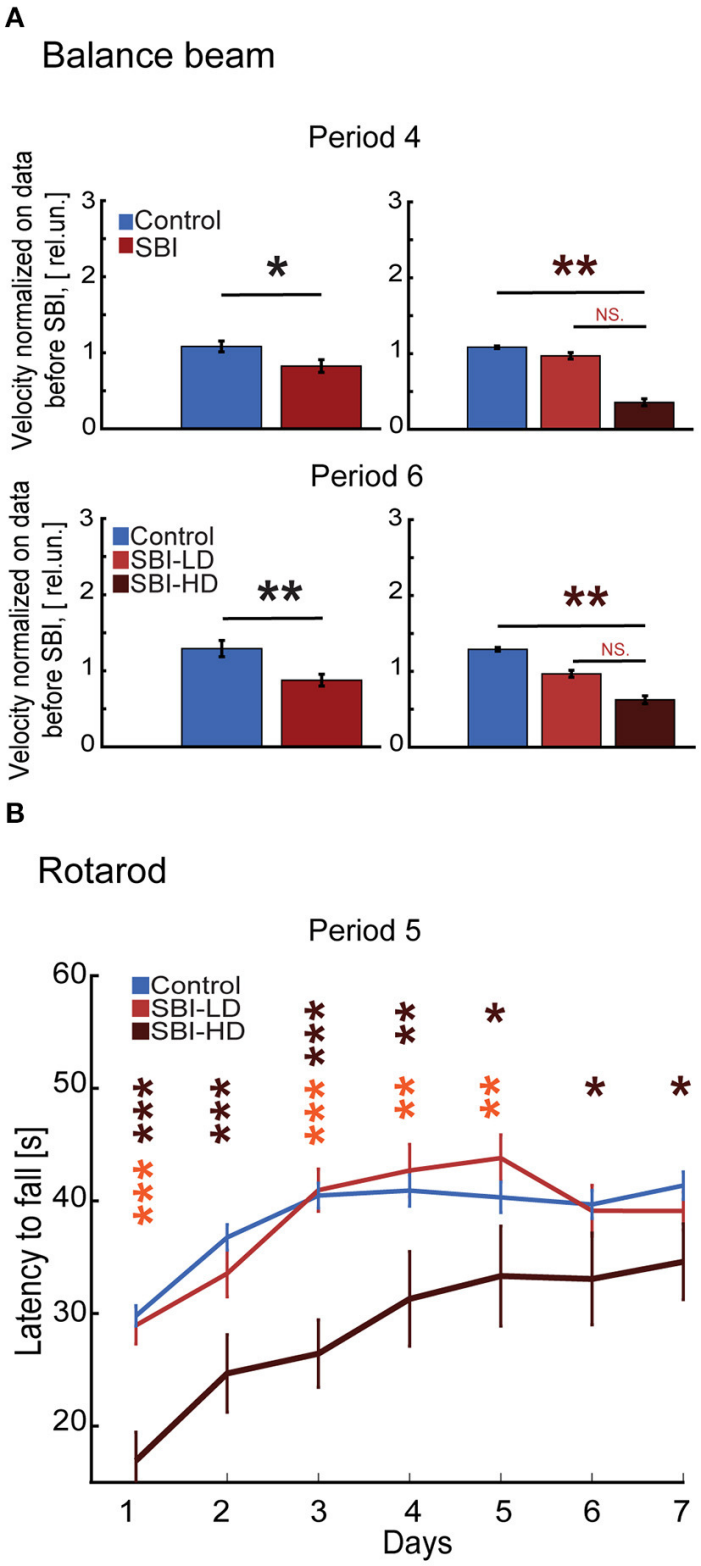
SBI chronically alters fine skill motor execution. **(A)** Illustrates the velocity reached by control (*n* = 22) and SBI (*n* = 30) while crossing the wooden beam a week (period 4) and 4 weeks (period 6) after SBI. Data were normalized to velocity reached before SBI. Data are shown in relative units (rel.un.). In addition, the data shows the balance beam performance when the SBI group was split into SBI-HD (*n* = 7) and SBI-LD (*n* = 8) and contrasted to control (*n* = 22). **(B)** The performance trajectories on a rotarod of control (*n* = 21), SBI-HD (*n* = 6), and SBI-LD (*n* = 7) after 3 weeks post-SBI (period 5). Data is presented in average durations (mean ± S.E.M.) of 5 trials per individual per day. We used the Kruskal–Wallis test and Dunn's with post hoc Bonferroni correction to determine the effect of blast injury on fine motor skills. *p < 0.05, **p < 0.01, ***p < 0.001, and non-significant statistical differences (N.S). Brown asterisks depict differences between SBI-HD and sham mice, and the orange asterisk display differences between SBI-HD and SBI-LD group.

Movement impairment is the most common long term problem reported by patients after traumatic brain injury (39%) (38). Impairments include issues with balance, coordination, ambulation, muscle tone (spasticity, paralysis, and weakness) and motor skills such as dexterity and writing (38, 39). We tested whether our SBI animal model displays these common sequelae associated with chronic post-injury stages. We tested SBI mice on the balance beam, a task requiring multisensory integration, motor coordination, and balance (40). During period 4 (Figure 1), sham mice performed markedly better than SBI mice (Mann–Whitney test, SBI: 0.83 ± 0.08; Ctrl: 1.08 ± 0.07 rel.un. *P* = 0.047). SBI-HD mice performed significantly worse [KW, *H*(2) = 11.66, MCDBC *P* = 0.003; Figure 4A] while SBI-LD were not different from control mice (*P* = 1.0). This difference in balance beam performance was also present during period 6 (SBI: 0.88 ± 0.08; Ctrl: 1.29 ± 0.1 rel.un. *P* = 0.003; Figure 4A-lower panel) with the SBI-HD group distinct from controls [KW, *H*(2) = 8.31, MCDBC *P* = 0.01]. The rotarod task, which assesses motor coordination and skill learning, showed similar results (Figure 4B). In this case, all animals were able to learn within the first 4 days when tested during the third week after blast treatment (semi-acute stage; period 5). However, the performance was significantly worse in SBI-HD mice (*P* < 0.001 in days 1–4 and *P* < 0.05 in days 6–7) than SBI-LD mice (*P* > 0.05) when these groups were compared to control mice. Interestingly, SBI-HD and SBI-LD were significantly different from each other (*P* < 0.01 days 1, 3–5; Figure 4B). In short, our data identified prominent motor deficits in our SBI model at chronic stages, with the worst symptoms observed in mice with an initial high delta/alpha ratio (SBI-HD).

### Dex improves sleep disturbances, motor, and cognitive behavior

**Figure 5:**
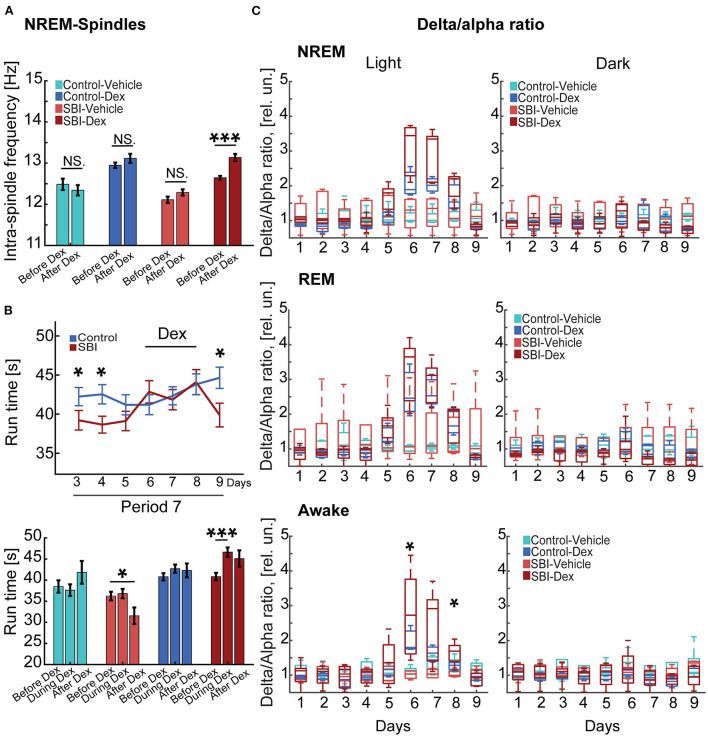
Dex-mediated sleep phase modulation ameliorates motor tasks. **(A)** Depicts changes in the intra-spindle frequency tracked for 24 h 3 days before and 3 days while injecting Dex injection (0.15 mg/Kg) in control mice injected with vehicle (*n* = 5), control mice injected with Dex (*n* = 4), SBI mice injected with vehicle (*n* = 4), and SBI mice injected with Dex (*n* = 5). **(B)** The upper panel shows the averaged (mean ± S.E.M.) run time on rotarod in seconds (s) of control (*n* = 21) and SBI mice (*n* = 27). In addition, we showed the run times 3 days before, during, and a day after Dex treatment (lower panel) including control mice injected with Dex (*n* = 11), control mice injected with vehicle (*n* = 10), SBI mice injected with Dex (*n* = 17) and SBI mice injected with vehicle (*n* = 11). **(C)** Displays box plots of the delta/alpha ratio scores obtained from control-vehicle (*n* = 5), control-Dex (*n* = 4), SBI-vehicle (*n* = 4), and SBI-Dex (*n* = 5) during the NREM, REM, and awake states. Each state was divided into 12 h of light and dark cycles. We applied Kruskal–Wallis and Dunn's test with Bonferroni correction to establish the effect of Dex on intra-spindle frequency, rotarod performance, and delta-alpha ratio. *p < 0.05, ***p < 0.001, and non-significant statistical differences (N.S).

Our data show motor and cognitive disabilities at chronic stages using tasks that are often influenced by sleep quality (12). Since increased spindle frequency and other features have been linked to memory formation, recall performance and skilled learning in humans (41–43) and rodents (44), we used a pharmacological approach to improve sleep spindle activity and then examined deficits in behavioral tasks. We treated mice with Dex, a drug that produces a state that closely resembles NREM sleep (stage 2) in humans and rodents, a stage when sleep spindles are prominent (16, 45). Importantly, subcutaneous injection of Dex produces minimal hemodynamic or oxygen saturation-secondary effects (46). We applied a drug concentration previously used in rodents (0.15 mg/Kg) (30) during period 7 (Figure 1) for three consecutive days. Injections were administered every day when the light cycle started. To determine the effect of Dex in our cohorts, we continuously assessed EEG/EMG activity starting 3 days before Dex injection, 3 days during administration of Dex treatment, and 1 day post-drug treatment. Our results showed a significant shift to higher intra-spindle frequencies in SBI mice injected with Dex (13.14 ± 00; Mann–Whitney test, *P* = 0.00005) compared to 3 days immediately before Dex. However, the intra-spindle frequency in other groups remained unmodified (control-vehicle, *P* = 0.42, control-Dex, *P* = 0.18, SBI-vehicle *P* = 0.06; Figure 5A). Also, since other studies (41, 42) found that increased intra-spindle frequency correlated with overnight consolidation of motor sequence skills and changes in activation in subcortical and cortico-motor regions, we assessed the motor behavior of animals 16 h after Dex injection. Although animals reached a plateau in motor performance (Figures 4B, 5B), Dex injection significantly improved motor performance in SBI mice with no significant differences compared to controls (Mann–Whitney test, day 6 SBI: 42.85 ± 1.43; Ctrl: 41.2 ± 1.27 s *P* = 0.69, day 7 SBI: 41.85 ± 1.3; Ctrl: 42.34 ± 1.15 s *P* = 0.43, and day 8 SBI: 44.1 ± 1.6 Ctrl: 43.81 ± 1.33 s *P* = 0.79; before Dex (*P* = 0.04; Figure 5B-upper panel). This improvement is due to the group injected with Dex (Figure 5B-lower panel). The drug-treated group showed significant differences in motor execution compared to the days preceding sedative treatment (before: 40.83 ± 0.9; and during Dex: 46.6 ± 1.0; Wilcoxon rank test Days 3–5 (period 7) vs. Dex days 6–8; *P* = 0.0003). In contrast, SBI mice injected with vehicle did not show significant changes in run times [before: 36.2 ± 1.2; and during Dex: 36.8 ± 1.1; Wilcoxon rank test Days 3–5 (period 7) vs. Dex days 6–8, *P* = 0.97]. After 3 days of Dex treatment, motor activity declined to a pre-treatment level in the SBI group (*P* = 0.02; Figure 5B) and the intra-spindle frequency shifted back to lower frequencies [12.38 ± 0.0; Wilcoxon rank test Days 3–5 (period 7) vs. day after Dex withdrawn (day 9) *P* = 0.9; Figure 5A]. Notably, while run times remained high a day after finishing treatment in SBI mice injected with Dex (45.1 ± 2; *P* = 0.05), SBI mice injected with vehicle worsened compared to records before the vehicle-treatment began (31.6 ± 2; *P* = 0.01; Figure 5B-lower panel). These data suggest that Dex acutely shifted intra-spindle oscillations to higher frequencies and concomitantly improved motor coordination in SBI mice.

Given the sedative effects of Dex, we expected an increase in the delta/alpha ratio (47). Indeed, we recorded an increase in the delta/alpha ratio (Figure 5C) during Dex injections (NREM-light cycle) compared to days immediately before injection. However, this increase was not significant in SBI or control mice under Dex treatment [KW, *H*(3) = 12.79, 12.92, 9.57; MCDBC SBI-Dex vs. SBI-Vehicle *P* > 0.05 days 6–8 and Control-Dex vs. Control-Vehicle *P* > 0.05 days 6–8]. Moreover, significant delta/alpha ratio changes were not observed in REM [KW, *H*(3) = 10.19, 9.75, 7.31; MCDBC SBI-Dex vs. SBI-Vehicle *P* > 0.05 and Control-Dex vs. Control-Vehicle *P* > 0.05]. In contrast, the awake state during the light cycle was significantly affected [KW, *H*(3) = 12.68, 7.64, 8.56, MCDBC, SBI-Dex vs. SBI-Vehicle day 6: *P* = 0.01, day 7: *P* = 0.08, day 8: *P* = 0.02 and Control-Dex vs. Control-Vehicle *P* > 0.3 days 6–8]. Importantly, the delta/alpha ratio did not show significant differences through the dark cycle (NREM: SBI-Dex vs. SBI-Vehicle *P* > 0.6, REM: SBI-Dex vs. SBI-Vehicle: *P* = 0.3, Awake: SBI-Dex vs. SBI-Vehicle *P* > 0.5 days 6–8; NREM: Control-Dex vs. Control-Vehicle *P* = 1.0, REM: Control-Dex vs. Control-Vehicle: *P* = 1.0, Awake: Control-Dex vs. Control-Vehicle *P* > 0.09 days 6–8). Moreover, the increased theta power noticed in SBI mice compared to controls during REM in period 5 and 7 diminished to control levels and we found no significant differences during the Dex injection (Wilcoxon rank test, Control vs. SBI Dex days 6–8: *P* > 0.68). Similarly, the alpha power differences observed before Dex (period 5 and 7) during the REM state (light cycle) in the SBI-HD group diminished compared to controls [KW, *H*(3) = 4.57, 3.07,4.76; MCDBC, Dex days 6–8 *P* > 0.09]. Collectively, these results show that Dex can acutely ameliorate sleep disruptions, shift intra-spindle activity to higher frequencies and increase motor execution in chronic injured mice without producing side effects.

**Figure 6:**
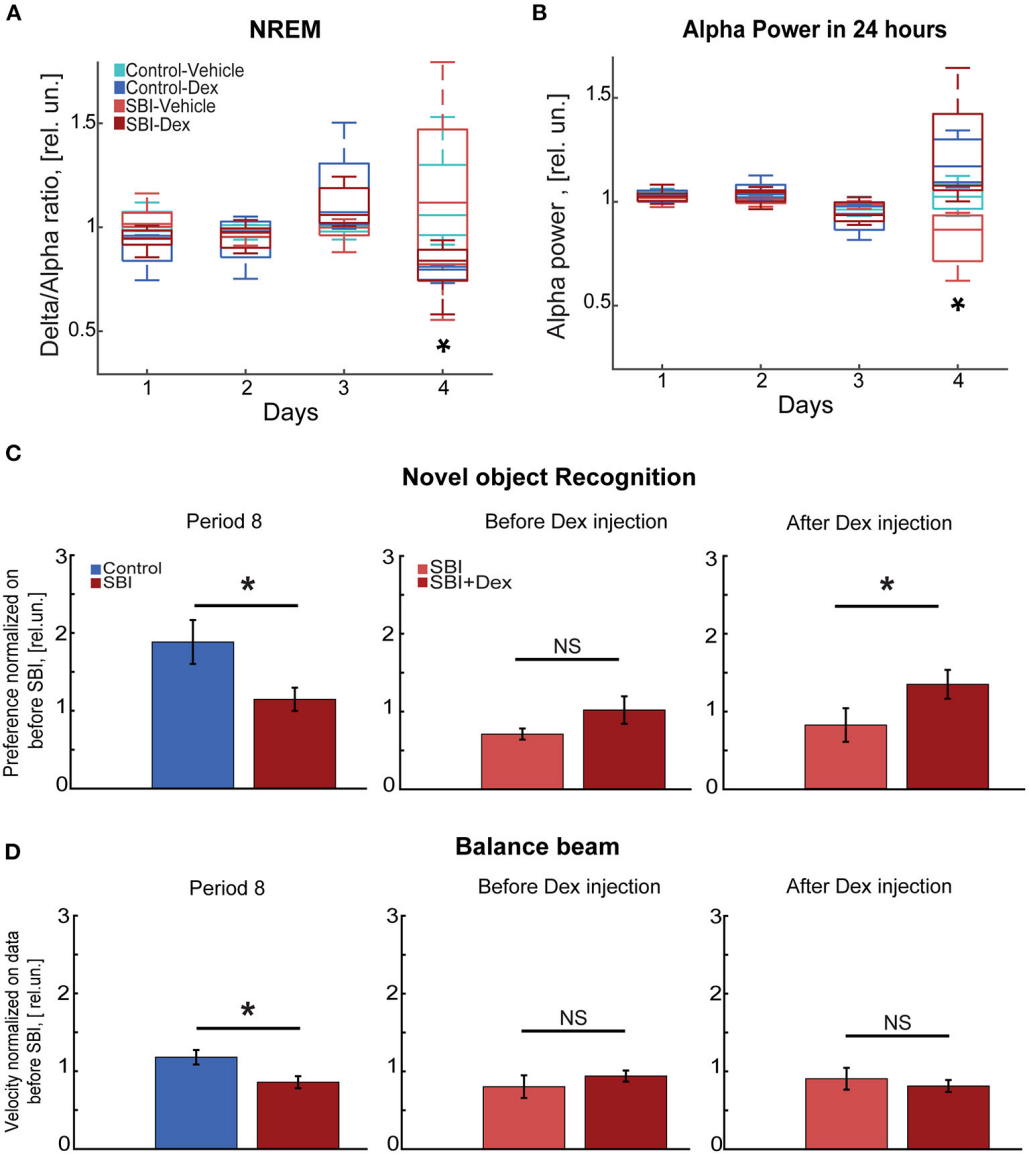
Cognitive tasks improve a week after Dex injection. **(A)** Delta/alpha ratio differences between SBI-Dex (*n* = 5), SBI-control (*n* = 4), Control-vehicle (*n* = 5), and Control-Dex (*n* = 4) during the NREM detected over 24 h and measured 3 days before SBI and compared to the ratio obtained a day after withdrawing Dex. Data were normalized to averaged ratio before SBI. **(B)** Alpha power measured 3 days before injury and compared to the alpha power measured a day after withdrawing Dex. Data were normalized to averaged alpha power before SBI. **(C)** Novel object task conducted at week 7 (period 8) in control (*n* = 22) and SBI (*n* = 28) mice, right side of the panel shows differences between SBI-vehicle (*n* = 12) and SBI-Dex (*n* = 16) groups observed before Dex injection (period 6) and 7 days post-Dex injection (period 8). Data were normalized to time spent exploring the novel object before injury and are shown in relative units (rel.un.). **(D)** Exhibits the velocity reached by control (*n* = 22) and SBI (*n* = 28) mice while performing on the balance beam at week 7 (period 8). The right side of the panel shows the differences between SBI-vehicle (*n* = 12) and SBI-Dex groups (*n* = 16) before Dex injection (period 6) and 7 days after Dex injection (period 8). Data were normalized to velocity reached before injury and shown in relative units (rel.un.). We employed the Mann–Whitney *U* test for two-group comparison to establish differences between control and SBI groups as well as in SBI groups with and without Dex injection while executing novel object recognition and balance beam tasks. *p < 0.05, and non-significant statistically differences (N.S).

Curiously, the delta/alpha ratio was restored to levels even lower than those observed prior to injury in SBI-Dex mice [Friedman test and Wilcoxon rank test with Bonferroni correction (FT&WRB), χ^2^(3) 9.24 day 1: *P* = 0.015, day 2: *P* = 0.061, day 3: *P* = 0.015; Figure 6A], but not in SBI-Vehicle group [FT&WRB, χ^2^(3) 3.6, day 1–3: *P* = 1.0, Figure 6A]. This is attributed to the increased alpha power observed after Dex withdrawal. Indeed, the alpha power reached values initially observed days before SBI [FT&WRB, χ^2^(3) 12.12, day 1: *P* = 0.89 and day 2: *P* = 1.0], whereas mice treated with vehicle alone did not [FT&WRB , χ^2^(3) 12.12; *P* = 0.04, day 1 and *P* = 0.02 day 2; Figure 6B]. Reduction in the alpha power in humans may indicate a disconnected state (48) and increased alpha power in REM is associated with memory, learning and attention (49). Thus, we studied whether Dex had a prolonged effect on cognitive and motor tasks (Figure 1; period 8). Our results show that animals performing the NOR test a week after Dex, showed significant improvement in performance compared to controls (SBI: 1.15 ± 0.15; Ctrl: 1.88 ± 0.28 rel.un. *P* = 0.01; Figure 6C) and SBI mice in periods 4 and 6 (Figure 3A). Notably, this improvement was attributed to the SBI population injected with Dex (Figure 6C). Indeed, the SBI-Dex group significantly improved compared to the SBI-vehicle in period 8 (Mann–Whitney test, SBI-Dex: 1.34 ± 0.19 SBI-vehicle: 0.81 ± 0.21 rel.un. *P* = 0.01). In contrast, balance beam performance by SBI-Dex treated mice remained unchanged (Figure 6D) compared to the SBI-vehicle group in period 8 (Mann–Whitney test, *P* = 0.01). Therefore, Dex had lasting effects on cognitive, but not motor, tasks (Mann–Whitney test, *P* = 0.72).

## Discussion

To our knowledge, this is the first study in which both sleep/wake cycle and behavior have been longitudinally studied for a period of 2 months following blast injury in mice. We evaluated mice 1 week before the blast injury and extended our analysis to ~6 weeks post-SBI. Our findings demonstrate an excessive sleep EEG pattern at acute stages post-SBI accompanied by substantial motor and cognitive impairment. We then observed a transition to more moderate and prolonged sleep/wake cycle disturbances, including changes in theta, alpha power, and disruptions of the NREM spindle amplitude and intra-spindle frequency associated with lasting motor and cognitive deficits. Direct modulation of the sleep phase using subcutaneous Dex restored theta and alpha power and reversibly improved intra-spindle frequency and motor execution in chronically injured mice. Moreover, Dex treatment ameliorated cognitive deficits a week post-injection.

We measured excessive sleep patterns in SBI mice during the acute post-injury stage compared to control mice finding (1) increased and sustained delta power across the awake, REM, and NREM states, (2) a prominent reduction in alpha and gamma power over 24 h, (3) increased total sleep time (REM+NREM), and (4) a significant reduction of the awake state. These concur with previous results where a diffuse blast wave (50) or an underwater blast (51) depressed both EEG frequency (dominance of 2–5 Hz activity) and amplitude in the minutes following post-traumatic injury in rats. Mice with a higher delta/alpha ratio (SBI-HD) had prominent, lasting sleep/wake cycle disturbances together with gross motor and cognitive task impairment. These results are consistent with previous findings in moderate and severe cases of traumatic brain injury in rodents (52–56) and humans (57, 58) in which hypersomnia was linked to greater traumatic brain injury severity. In contrast, mice with a low delta/alpha ratio (SBI-LD) manifested long-term milder EEG, motor, and cognitive disruptions. Although in mild trauma EEG changes remain variable across traumatic brain injury models (4), all together, our findings suggest the SBI-LD group showed mild post-injury symptoms. Notably, the delta/alpha ratio can categorize and predict outcomes after stroke in rodents (59) and humans (8, 60, 61). Indeed, this biomarker facilitated analysis of recovery trajectories in our model.

Furthermore, lasting EEG disturbances at semi-acute and chronic stages, such as low alpha power in REM, increased theta power in REM and awake states, and modifications in the spindle features were evident during the third and fifth weeks after blast injury. These data are unprecedented: previous EEG records from blast-injured rodent models were limited to 30 min following brain trauma (50, 51).

Intriguingly, during the semi acute stage (period 5), SBI-LD animals showed a significant increase of theta power during the awake state compared to the control group. However, this change did not persist in period 7. Increased theta power has been associated with the processing of novel information (62), enhanced perceptive-cognitive processing (63) and physical training (64). We speculate that the increased theta power in SBI-LD mice is a compensatory event that instigated the learning and motor performance improvement observed in this group. Moreover, these lasting EEG effects mimicked adverse outcomes occurring months or years after brain trauma in humans (37). For instance, inflammation was previously shown to significantly affect thalamic nuclei in humans (65) and rodents (37) long after injury. Chronic inflammation produces low calcium levels and smaller spontaneous inhibitory postsynaptic currents (IPSCs) in rodent GABAergic reticular thalamic neurons (37). As a result, spindle activity generated by this thalamic area (12) during NREM sleep is disrupted, resulting in less spindle stability and sleep disruptions. These EEG characteristics are commonly found in traumatic brain injured patients experiencing impaired learning (66).

Similarly, we observed prolonged cognitive deficits in our blast injury model. A week after injury, spatial recognition was impaired in the NOR and Y maze tasks, consistent with low cognitive performance in other blast injury models at seven days post-SBI in the Y maze (67, 68) and other cognitive tasks (69–71). Our study found prolonged impairment in NOR during the fourth week after SBI, consistent with poor memory performance impairment thirty days post-injury in the Barnes maze (70, 72). Interestingly, SBI mice did not show lasting impairment in the Y-maze task. Improved spatial reference memory might be related to restoration of sleep structure during period 3. It is known that prominent delta power disrupts NREM sleep spindles and, therefore, spatial memory tasks (73). Returning delta power to baseline conditions might improve spatial memory consolidation.

Our findings regarding balance beam performance align with other studies in blast-injured rodents where motor performance was significantly diminished a week after injury (68–70, 74, 75). Intriguingly, balance beam execution during the fourth week (period 6) and rotarod during the fifth week (period 7) post-injury persistently showed motor deficits in our SBI-HD mice. These findings contrast with studies where rotarod performance spontaneously recovers 2 weeks post-injury (68–70, 74, 75).

We found that Dex treatment at chronic stages shifted intra-spindle oscillations to higher frequencies with concomitant motor performance improvement in SBI mice. Indeed, changes in spindles have been associated with clinical recovery in traumatic brain injury patients (14). In particular, a reduction in frequency and amplitude in cortical spindles was seen at subacute stages. While subjects transition from the subacute to the chronic stage, spindle activity improves together with cognitive function, perceptual organization, and working memory (14). Our data showed, for the first time that a pharmacological intervention can reverse persistent changes in intra-spindle frequencies associated with impaired motor behavior in mice. Remarkably, dexmedetomidine enhanced motor recovery during chronic stages compared to the deterioration seen in our SBI-vehicle group. Although we speculate this motor recovery is associated with reduced sleep disturbances, we are aware that Dex may also improve chronic neuropathic pain (76). Future studies will distinguish these two components.

Moreover, prolonged Dex effects were recorded a day after ending treatment: delta/alpha ratio were restored to levels lower than pre-SBI and alpha and theta power returned to baseline levels. Memory also improved days after ending Dex treatment. Indeed, Dex infusion can chronically improve cognitive function and quality of life in 3-year survivors (77, 78).

Since spindle oscillations provoke a massive Ca^2+^entry into the spindling cortical cells and could set the stage for long-term plastic synaptic changes (12, 79), we propose that modulation of the sleep phase using Dex may produce an increase in gray matter in cortical and subcortical areas. Indeed, studies combining EEG sleep recordings and high-resolution structural brain imaging have shown that fast spindles increase gray matter in the hippocampus (80). This outcome may contribute to a more effective transfer of hippocampal-dependent declarative memories to the cortex (81). Here, we have demonstrated that dexmedetomidine significantly shifts the intra-spindle frequency to higher frequencies (similar range to Saletin et al.) and ameliorates cognitive tasks and motor performance in a chronic phase. Thus, it is feasible that this mechanism occurs in our animal model. We will explore this intriguing mechanism in future studies. It is also likely that anti-inflammatory and immunomodulatory (82–84) effects of Dex contribute to improving EEG activity and cognition days after Dex treatment. However, further studies are necessary to directly establish the effect of Dex at chronic stages on neuroinflammation and EEG modulation.

Prolonged (>24 h) and continuous high-dose Dex can cause initial transient hypertension through peripheral a_2b_-receptors followed by hypotension due to action on central a_2a_-receptors. However, we subcutaneously injected Dex at 24-h intervals. Although we did not measure blood pressure in our animals, others have shown that subcutaneous treatment reduces Dex hemodynamic secondary effects, oxygen saturation remains unchanged, and sedative effects are less abrupt than intravenous administration (46).

Although continuous Dex infusion may lead to tolerance and tachyphylaxis (85), our daily evaluations confirmed high motor activity during successive injections. Similarly, improved memory performance remained a week after Dex treatments indicating that tolerance or tachyphylaxis were not observed with our Dex injection protocol. In addition, although Dex plasma concentrations are 31% lower than those reached using intravenous treatment (46), the concentration used in our experiments (0.1 mg/Kg) was sufficient to modulate intra-spindle frequency and improve motor behavior. In contrast, Chamadia et al. (86) found that orally administered Dex increased the duration of NREM stage 2 sleep and spindle density, but saw no motor performance improvement compared to placebo. The authors suggested that a residual drug effect might have impaired motor performance. Thus, SC Dex may be a more feasible alternative for chronically injured patients.

This study has several limitations. First, we used a single Dex dose and did not, therefore, establish the minimal Dex concentration necessary to produce cortical and behavioral improvement in our chronically injured mice. Moreover, we recorded EEG activity 1 day after withdrawing Dex. Thus, we cannot determine the longer term persistence of the Dex effect on abnormal and lasting EEG patterns. One condition examined in this study is the presence of cognitive deficits after injury. However, as we previously described (18), our female SBI model does not show significant impairment in cognitive tasks. Nevertheless, we acknowledge that SBI females may also exhibit sleep disturbances, a factor that we will explore in future studies. Lastly, our approach limits establishment of a direct association between lasting EEG changes and cognitive improvement, since EEG recordings and cognitive tasks were obtained in different weeks. Further analysis will allow the effects of Dex to be tested in human studies.

## Conclusions

We found that acute post-SBI models exhibit excessive sleep accompanied by significant motor and cognitive impairment. Sleep-associated EEG features improved several weeks after SBI. However, several abnormal characteristics and impaired motor and cognitive behavior persisted. Importantly, we showed that nocturnal subcutaneous injection of Dex for several days improved persistent abnormal EEG features, motor and cognitive execution in blast-injured mice. We postulate that subcutaneous Dex treatment may be a feasible approach to improving sleep anomalies as well as impaired motor and cognitive function in chronically injured patients.

## Data availability statement

The raw data supporting the conclusions of this article will be made available by the authors, without undue reservation.

## Ethics statement

The animal study was reviewed and approved by the Weill Cornell Medicine IACUC (Protocol No. 2016-0054).

## Author contributions

YB: data curation (lead), formal analysis (lead), investigation (equal), software (lead), validation (equal), visualization (equal), and writing-review and editing (supporting). NS: conceptualization (equal), methodology (equal), validation (equal), and review and editing (equal). DC: conceptualization (lead), funding acquisition (lead), investigation (lead), methodology (lead), resources (lead), supervision (lead), validation (lead), visualization (lead), writing-original draft (lead), and review and editing (lead). All authors contributed to the article and approved the submitted version.

## Funding

This work was supported by the Nancy M. and Samuel C. Fleming Research Scholar awarded to DC.

## Conflict of interest

The authors declare that the research was conducted in the absence of any commercial or financial relationships that could be construed as a potential conflict of interest.

## Publisher's note

All claims expressed in this article are solely those of the authors and do not necessarily represent those of their affiliated organizations, or those of the publisher, the editors and the reviewers. Any product that may be evaluated in this article, or claim that may be made by its manufacturer, is not guaranteed or endorsed by the publisher.

## Abbreviations

Dex: dexmedetomidine
EMG: electromyogram
FT&WRB: friedman test and wilcoxon rank test with Bonferroni correction
MCDBC: multiple comparisons using Dunn's test with Bonferroni correction
NREM: non-rapid eye movements
SBI: secondary blast injury
SBI-LD: group of animals with no changes in Delta/Alpha ratio compared to controls after secondary blast injury
SBI-HD: group of animals with increased Delta/Alpha ratio after secondary blast injury
SC: subcutaneous.
