# Supplementary material for: Dexmedetomidine-mediated sleep phase modulation ameliorates motor and cognitive performance in a chronic blast-injured mouse model
Source: Front Neurol. 2022 Nov 1;13:1040975. doi: 10.3389/fneur.2022.1040975 (PMC9663850; doi:10.3389/fneur.2022.1040975)
Supplement: Supplementary file 1 [file Presentation_1.PDF]

Supplementary Fig 1. Blast injured mice show sleep disturbances during the semi-acute stage post-trauma. (**A**)Delta alpha ratio differences between SBI-HD (n=6), SBI-LD (n=7), and sham mice (n=10) in NREM, REM, and Wake states during the third week after injury (**B**) NREM, REM, and awake episode duration over 24 hours in SBI-HD (n=6), SBI-LD(n=7) and sham mice(n=10) and normalized to averaged episode duration before SBI. (**C**) Average peak domain amplitude (mean±S.E.M) in microvolts of the detected spindles in 24-hour periods in SBI-HD (n=6), SBI-LD (n=7), and sham(n=10) normalized to the averaged peak found before SBI in relative units (rel. un.) (**D**) Averaged intra-spindle frequency distribution (mean±S.E.M) in detected spindles during 24 hours in SBI-HD, SBI-LD, and control mice three weeks after inflicting SBI. Kruskal Wallis test with Bonferroni correction, Trend (T)= $P>0.05$ to 0.09, *p=0.05, ***p*=0.01, ****p*=0.001. Brown asterisks depict differences between SBI-HD and sham mice, and the orange asterisk display differences between SBI-HD and SBI-LD group.

Supplementary Fig 2. Blast injured mice display sleep disturbances at a chronic stage post-trauma. (**A**)Delta alpha ratio differences between SBI-HD (n=5), SBI-LD (n=6), and sham mice (n=9) in NREM, REM, and Wake states during the fifth week after injury (**B**) NREM, REM, and awake episode duration over 24 hours in SBI-HD (n=5), SBI-LD(n=6) and sham mice(n=9) and normalized to averaged episode duration before SBI. (**C**) Average peak domain amplitude (mean±S.E.M) in microvolts of the detected spindles in SBI-HD, SBI-LD, and sham mice during 24-hour periods and normalized to the averaged peak found before SBI represented in relative units (rel. un.). (**D**) Averaged intra-spindle frequency distribution (mean±S.E.M) in detected spindles during 24-hour periods in SBI-HD, SBI-LD, and control five weeks after causing SBI. Kruskal Wallis test with Bonferroni correction, Trend (T)= $P>0.05$ to 0.09, *p=0.05, ***p*=0.01, ****p*=0.001. Carmine asterisks represent differences between sham and SBI-LD, brown asterisks depict differences between SBI-HD and sham mice, and the orange asterisk display differences between SBI-HD and SBI-LD groups.

Semi-acute stage post-TBI
period 5

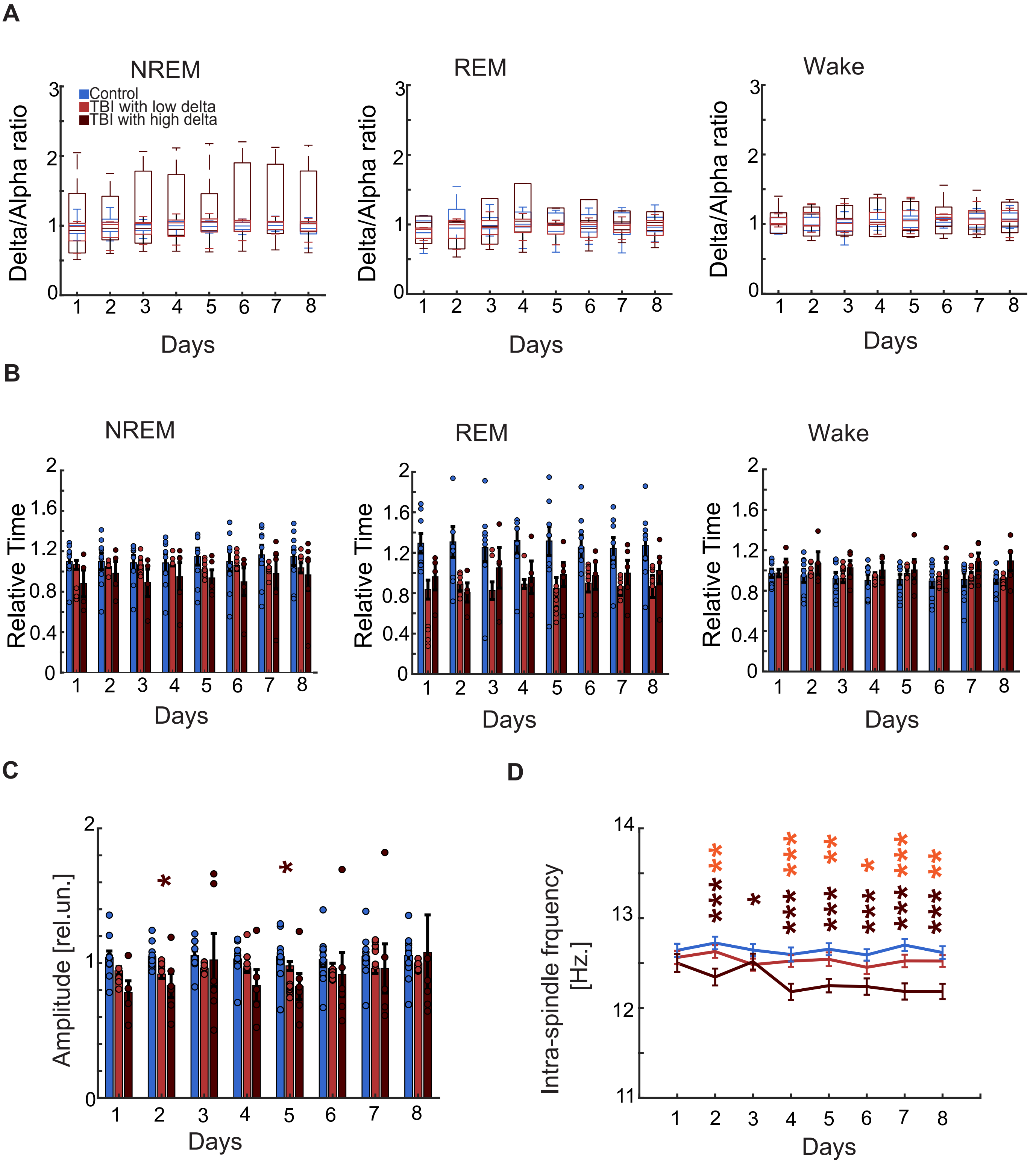

Supplementary Figure 1

Chronic stage post-TBI
period 7

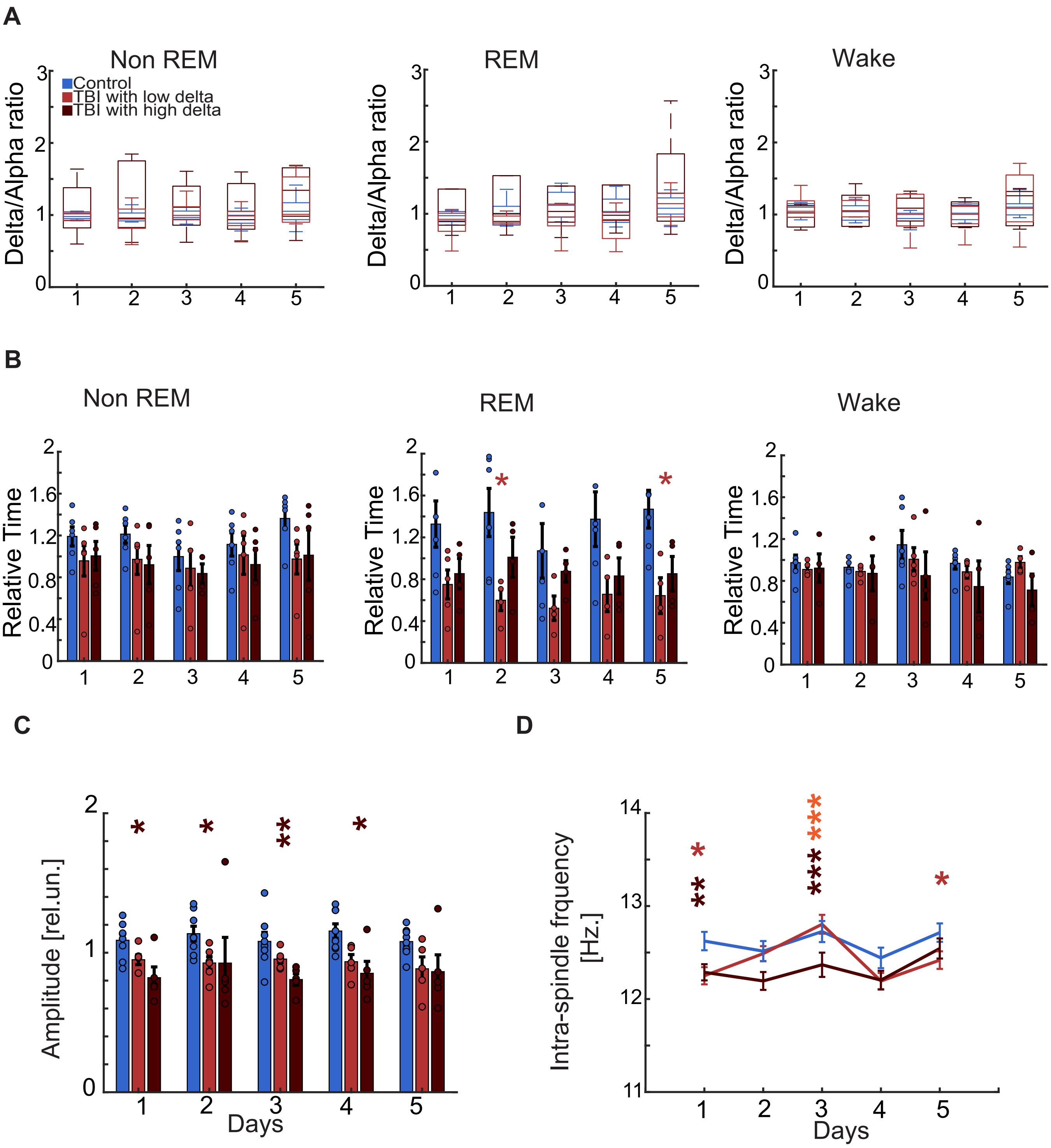

Supplementary Figure 2

Supplementary Table1. Averaged and standard error (±SE) power frequency changes in awake, NREM and REM states were quantified 24 hours before (Period 2; days 1-3) and after causing secondary blast injury (SBI; acute stage) in male mice (Period 3; SBI-6th day). *P* values (*P* val.) were obtained by comparing power frequencies between control and SBI mice.

**Condition**	**Period 2**			**Period 3**					
**NREM**	1	2	3	**SBI**	2	3	4	5	6
**Delta power within 24 hours**									
Sham	1.003	0.999	0.998	0.969	1.025	1.004	1.013	1.026	1.053
±SE	0.009	0.01	0.011	0.035	0.024	0.011	0.017	0.011	0.026
SBI	1.006	1.001	0.993	1.137	1.204	1.244	1.162	1.094	1.01
±SE	0.005	0.005	0.006	0.038	0.053	0.059	0.056	0.05	0.041
P val.	0.909	0.449	0.763	**0.003**	**0.027**	**0.001**	**0.019**	0.863	0.132
**Theta power within 24 hours**									
Sham	0.998	1.005	0.997	1.002	0.991	0.995	0.985	1	0.982
±SE	0.009	0.008	0.004	0.015	0.01	0.012	0.027	0.013	0.018
SBI	0.996	1.002	1.002	0.948	0.895	0.918	0.963	0.974	0.996
±SE	0.003	0.002	0.004	0.022	0.05	0.032	0.028	0.029	0.026
P val	0.536	0.224	0.414	0.156	0.301	0.082	0.505	0.98	0.35
**Alpha power within 24 hours**									
Sham	1.001	1.01	0.99	0.985	0.99	1.007	0.967	0.984	0.998
±SE	0.005	0.008	0.009	0.022	0.018	0.012	0.031	0.019	0.022
SBI	0.997	1.016	0.987	0.893	0.791	0.813	0.867	0.903	0.978
±SE	0.006	0.007	0.009	0.028	0.052	0.043	0.044	0.04	0.037
P val.	0.566	0.347	1	**0.031**	**0.006**	0	0.145	0.109	0.5
**Beta power within 24 hours**									
Sham	1	0.999	1.001	1.021	0.98	0.989	0.985	0.971	0.956
±SE	0.009	0.008	0.012	0.027	0.013	0.011	0.013	0.015	0.016
SBI	0.997	0.999	1.004	0.918	0.843	0.858	0.87	0.925	0.978
±SE	0.006	0.005	0.008	0.023	0.037	0.055	0.042	0.04	0.032
P val	0.731	0.663	0.366	**0.008**	**0.002**	0	**0.032**	0.336	0.161
**Gamma power within 24 hours**									
Sham	0.981	0.951	0.99	1.115	0.914	0.971	0.915	0.907	0.859
±SE	0.018	0.016	0.015	0.06	0.038	0.033	0.024	0.025	0.036
SBI	0.965	0.933	1.005	0.926	0.778	0.783	0.8	0.9	0.944
±SE	0.01	0.021	0.017	0.041	0.043	0.045	0.049	0.058	0.029
P val.	0.142	0.721	0.3	0.026	**0.039**	**0.009**	**0.07**	0.362	0.101
**Condition**	**Period 2**			**Period 3**					
**REM**	1	2	3	**SBI**	2	3	4	5	6
**Delta power within 24 hours**									
Sham	1.006	0.999	0.996	0.933	1.004	1.01	0.988	1.016	1.04
±SE	0.016	0.014	0.023	0.032	0.025	0.019	0.021	0.033	0.059

SBI	0.999	0.996	1.005	1.3	1.411	1.447	1.237	1.135	0.982
±SE	0.007	0.006	0.007	0.087	0.13	0.13	0.117	0.093	0.067
P val.	0.506	0.63	0.168	0	**0.002**	**0.01**	**0.036**	0.639	0.097
**Theta power within 24 hours**									
Sham	1	1.007	0.993	0.99	0.982	0.977	0.971	0.994	0.991
±SE	0.008	0.006	0.008	0.011	0.011	0.009	0.024	0.011	0.021
SBI	1.003	0.995	1.002	0.927	0.863	0.928	1.008	1.012	1.057
±SE	0.004	0.006	0.007	0.023	0.055	0.04	0.038	0.037	0.034
P val.	0.872	0.191	0.699	0.111	0.158	0.452	0.289	0.312	0.194
**Alpha power within 24 hours**									
Sham	1.01	1.011	0.98	0.987	1.012	1.02	0.978	0.975	0.973
±SE	0.009	0.006	0.009	0.023	0.023	0.014	0.025	0.018	0.018
SBI	1	1.022	0.979	0.871	0.836	0.865	0.91	0.896	0.929
±SE	0.009	0.009	0.008	0.031	0.055	0.04	0.039	0.031	0.026
P val.	0.697	0.396	0.931	**0.035**	**0.013**	**0.005**	0.388	0.175	0.194
**Beta power within 24 hours**									
Sham	0.995	1.002	1.002	1.018	1.003	1.005	1.005	1.004	1.009
±SE	0.012	0.006	0.009	0.011	0.009	0.012	0.014	0.017	0.019
SBI	0.991	1.008	1.001	0.991	0.945	0.95	0.922	0.975	0.98
±SE	0.006	0.007	0.009	0.02	0.053	0.059	0.027	0.024	0.023
P val.	0.801	0.836	0.796	0.389	**0.043**	**0.01**	**0.01**	0.267	0.254
**Gamma power within 24 hours**									
Sham	0.974	0.938	0.975	1.062	0.938	0.998	0.975	0.971	0.968
±SE	0.019	0.021	0.021	0.031	0.015	0.026	0.028	0.039	0.01
SBI	0.983	0.971	0.979	0.946	0.75	0.77	0.807	0.894	1.022
±SE	0.01	0.01	0.01	0.033	0.042	0.045	0.04	0.03	0.026
P val.	0.923	0.178	0.794	**0.074**	**0.003**	**0.002**	**0.003**	0.133	0.152
**Condition**	**Period 2**			**Period 3**					
**Wake**	1	2	3	**SBI**	2	3	4	5	6
**Delta power within 24 hours**									
Sham	0.988	1.044	0.971	0.954	1.008	1.052	1.046	1.11	1.189
±SE	0.012	0.018	0.012	0.024	0.032	0.037	0.055	0.064	0.077
SBI	0.992	1.029	0.979	1.225	1.39	1.447	1.239	1.095	1.008
±SE	0.012	0.013	0.013	0.054	0.1	0.112	0.093	0.073	0.055
P val.	0.536	0.506	0.897	**0.001**	**0.008**	**0.016**	0.109	0.474	**0.011**
**Theta power within 24 hours**									
Sham	1	0.999	1	1.008	1.002	0.991	0.959	0.986	0.973
±SE	0.007	0.007	0.007	0.014	0.012	0.02	0.032	0.016	0.013
SBI	0.998	1.004	0.998	0.986	0.915	0.968	1.032	1.047	1.036
±SE	0.003	0.006	0.007	0.027	0.058	0.044	0.033	0.038	0.037
P val.	0.982	0.477	0.966	0.966	0.605	0.851	0.098	0.246	0.276
**Alpha power within 24 hours**									
Sham	1.018	0.975	1.006	1.051	1.065	0.997	0.965	0.968	0.914
±SE	0.011	0.018	0.011	0.035	0.027	0.026	0.04	0.032	0.04

SBI	1.022	0.962	1.016	0.843	0.749	0.797	0.842	0.875	0.879
±SE	0.01	0.013	0.013	0.035	0.06	0.041	0.036	0.032	0.016
P val.	1	0.324	0.699	**0.001**	**0**	**0.002**	**0.025**	**0.041**	0.194
**Beta power within 24 hours**									
Sham	0.998	1	1.002	0.98	0.964	0.967	0.947	0.948	0.968
±SE	0.01	0.006	0.007	0.014	0.013	0.013	0.019	0.029	0.03
SBI	0.993	1.01	0.997	0.98	0.902	0.934	0.962	1.004	1.041
±SE	0.006	0.005	0.008	0.03	0.057	0.059	0.044	0.041	0.038
P val.	0.506	0.302	0.667	0.863	0.132	0.24	0.416	0.175	0.02
**Gamma power within 24 hours**									
Sham	0.997	0.962	0.994	1.005	0.935	0.955	0.955	0.907	0.901
±SE	0.014	0.017	0.006	0.01	0.009	0.02	0.033	0.033	0.034
SBI	0.978	0.968	0.992	0.825	0.676	0.577	0.74	0.882	0.985
±SE	0.007	0.01	0.01	0.026	0.04	0.04	0.051	0.043	0.039
P val.	0.055	0.975	1	0	0	0	**0.008**	0.911	**0.025**

Supplementary table 2. Averaged and standard error (±SE) power frequency changes in awake, NREM and REM states were quantified during 24 hours in Period 5 (days 1-8). *P* values (*P* val.) were obtained comparing power frequencies between control, SBI-HD and SBI-LD mice.

**Condition NREM**	**Period 5**							
	1	2	3	4	5	6	7	8
**Delta power within 24 hours**								
Sham	1.005	1.004	1.01	1.008	1.011	1.006	1.015	0.994
±SE	0.033	0.029	0.019	0.023	0.026	0.024	0.023	0.026
SBI-LD	0.938	0.957	0.979	0.99	0.995	0.992	0.993	0.994
±SE	0.045	0.041	0.038	0.037	0.036	0.027	0.025	0.023
SBI-HD	1.011	0.988	1.064	1.049	1.054	1.089	1.09	1.085
±SE	0.081	0.071	0.103	0.094	0.083	0.094	0.095	0.095
***P* val. Sham vs SBI-HD**	1.000	1.000	1.000	1.000	1.000	1.000	1.000	1.000
***P* val. Sham vs SBI-LD**	1.000	1.000	1.000	1.000	1.000	1.000	1.000	1.000
***P* val. SBI-LD vs SBI-HD**	1.000	1.000	1.000	1.000	1.000	1.000	1.000	1.000
**Theta power within 24 hours**								
Sham	0.973	0.978	0.982	0.983	0.989	0.982	0.989	0.996
±SE	0.01	0.009	0.009	0.009	0.009	0.01	0.008	0.008
SBI-LD	1.015	1.004	1.003	1.002	0.992	1.002	0.997	0.998
±SE	0.016	0.013	0.017	0.018	0.014	0.012	0.012	0.013
SBI-HD	1.013	1.032	0.981	0.98	0.987	0.959	0.96	0.961
±SE	0.035	0.032	0.052	0.045	0.035	0.047	0.046	0.05
***P* val. Sham vs SBI-HD**	1	0.967	1	1	1	1	1	1
***P* val. Sham vs SBI-LD**	0.359	1	1	1	1	1	1	1
***P* val. SBI-LD vs SBI-HD**	1	1	1	1	1	1	1	1
**Alpha power within 24 hours**								
Sham	1.004	1.001	1.013	1.021	1.019	1.015	1.018	1.035
±SE	0.031	0.021	0.018	0.018	0.018	0.021	0.02	0.023
SBI-LD	1.049	1.029	1.012	1.01	1.01	0.998	0.999	1.005
±SE	0.03	0.027	0.019	0.025	0.026	0.018	0.02	0.017
SBI-HD	0.949	0.954	0.923	0.937	0.944	0.925	0.935	0.949
±SE	0.109	0.095	0.107	0.106	0.084	0.091	0.087	0.091
***P* val. Sham vs SBI-HD**	1	1	0.617	1	0.949	0.892	1	0.82
***P* val. Sham vs SBI-LD**	1	1	1	1	1	1	1	0.932
***P* val. SBI-LD vs SBI-HD**	1	0.684	1	1	1	1	1	1
**Beta power within 24 hours**								

Sham	1.038	1.042	1.033	1.039	1.021	1.042	1.024	1.038
±SE	0.052	0.045	0.036	0.042	0.042	0.042	0.037	0.041
SBI-LD	1.059	1.05	1.031	1.02	1.023	1.014	1.026	1.018
±SE	0.048	0.041	0.031	0.033	0.034	0.02	0.021	0.022
SBI-HD	1.028	1.042	1.032	1.024	0.986	1.005	1	1
±SE	0.112	0.102	0.107	0.097	0.088	0.087	0.09	0.088
***P* val. Sham vs SBI-HD**	1	1	1	1	1	1	1	1
***P* val. Sham vs SBI-LD**	1	1	1	1	1	1	1	1
***P* val. SBI-LD vs SBI-HD**	1	1	1	1	1	1	1	1
**Gamma power within 24 hours**								
Sham	0.971	0.993	0.94	0.935	0.902	0.921	0.932	0.935
±SE	0.035	0.046	0.028	0.039	0.036	0.054	0.034	0.035
SBI-LD	1.003	1.023	0.966	0.973	1.007	1.011	1.02	0.988
±SE	0.023	0.024	0.041	0.028	0.041	0.034	0.044	0.034
SBI-HD	1.017	1.046	0.974	0.967	0.992	1	0.95	0.952
±SE	0.089	0.105	0.082	0.042	0.084	0.057	0.08	0.068
***P* val. Sham vs SBI-HD**	0.48	0.672	0.917	1	0.446	0.624	1	1
***P* val. Sham vs SBI-LD**	1	1	1	1	0.59	0.618	0.297	1
***P* val. SBI-LD vs SBI-HD**	0.859	0.781	1	1	1	1	1	1
**Condition REM**	**Period 5**							
	1	2	3	4	5	6	7	8
**Delta power within 24 hours**								
Sham	1.028	1.033	1.001	1.008	1.01	0.986	0.992	0.971
±SE	0.062	0.055	0.044	0.042	0.05	0.04	0.048	0.048
SBI-LD	0.908	0.944	0.954	0.96	0.954	0.96	0.972	0.956
±SE	0.047	0.042	0.041	0.041	0.042	0.035	0.035	0.042
SBI-HD	1.023	0.979	1.067	1.042	1.05	1.094	1.066	1.056
±SE	0.153	0.134	0.175	0.18	0.161	0.167	0.152	0.166
***P* val. Sham vs SBI-HD**	0.33	0.186	1	1	1	1	1	1
***P* val. Sham vs SBI-LD**	0.321	0.983	1	1	1	1	1	1
***P* val. SBI-LD vs SBI-HD**	1	1	1	1	1	1	1	1
**Theta power within 24 hours**								
Sham	0.946	0.941	0.967	0.974	0.966	0.976	0.984	0.987
±SE	0.023	0.022	0.018	0.02	0.019	0.02	0.018	0.021
SBI-LD	1.003	0.994	1.009	1.015	1.028	1.021	1.015	1.033
±SE	0.017	0.015	0.014	0.015	0.027	0.031	0.026	0.037
SBI-HD	1.031	1.053	1.042	1.06	1.025	1.004	1.014	1.023
±SE	0.089	0.075	0.091	0.097	0.074	0.07	0.069	0.065

***P* val. Sham vs SBI-HD**	0.398	0.428	0.577	0.098	0.219	0.723	0.961	0.5
***P* val. Sham vs SBI-LD**	0.684	0.453	0.704	0.748	0.326	0.516	1	1
***P* val. SBI-LD vs SBI-HD**	1	1	1	0.91	1	1	1	1
**Alpha power within 24 hours**								
Sham	0.982	0.984	1.008	0.998	1.008	0.999	1.009	1.013
±SE	0.028	0.022	0.021	0.018	0.016	0.015	0.02	0.018
SBI-LD	1.055	1.03	1.005	0.996	1.015	1.014	1.013	0.999
±SE	0.026	0.031	0.024	0.017	0.017	0.02	0.018	0.016
SBI-HD	0.864	0.858	0.842	0.861	0.884	0.887	0.88	0.888
±SE	0.072	0.066	0.074	0.084	0.076	0.071	0.062	0.062
***P* val. Sham vs SBI-HD**	0.255	0.157	**0.031**	0.147	0.147	0.211	**0.065**	**0.046**
***P* val. Sham vs SBI-LD**	0.53	1	1	1	1	1	1	1
***P* val. SBI-LD vs SBI-HD**	**0.016**	**0.024**	0.073	0.166	0.063	0.103	0.069	0.195
**Beta power within 24 hours**								
Sham	1.059	1.08	1.063	1.058	1.054	1.068	1.056	1.051
±SE	0.054	0.05	0.039	0.043	0.04	0.04	0.038	0.04
SBI-LD	1.042	1.051	1.016	0.998	1.008	1.013	1.016	0.995
±SE	0.036	0.031	0.019	0.014	0.025	0.021	0.014	0.022
SBI-HD	1.055	1.047	1.057	1.019	1.016	1.041	1.064	1.063
±SE	0.057	0.043	0.068	0.055	0.033	0.038	0.058	0.054
***P* val. Sham vs SBI-HD**	1	1	1	1	1	1	1	1
***P* val. Sham vs SBI-LD**	1	1	1	1	1	1	1	1
***P* val. SBI-LD vs SBI-HD**	1	1	1	1	1	1	1	0.855
**Gamma power within 24 hours**								
Sham	1.002	1.013	0.99	0.949	0.974	0.981	0.951	0.945
±SE	0.029	0.034	0.027	0.024	0.056	0.042	0.044	0.042
SBI-LD	0.982	0.98	0.989	0.966	0.935	0.951	0.948	0.936
±SE	0.037	0.013	0.028	0.021	0.018	0.031	0.03	0.035
SBI-HD	0.986	0.991	0.908	0.916	0.986	0.962	0.938	0.994
±SE	0.03	0.019	0.078	0.025	0.039	0.047	0.013	0.054
***P* val. Sham vs SBI-HD**	1	1	1	0.896	1	1	1	1
***P* val. Sham vs SBI-LD**	1	0.775	1	1	0.532	1	1	1
***P* val. SBI-LD vs SBI-HD**	1	1	0.922	0.82	0.938	1	1	0.942
**Condition Wake**	**Period 5**							
	1	2	3	4	5	6	7	8
**Delta power within 24 hours**								

Sham	1.054	1.034	1.011	0.988	0.994	0.985	0.997	1.008
±SE	0.069	0.049	0.05	0.045	0.049	0.048	0.05	0.049
SBI-LD	0.999	1.007	0.998	1.02	1.025	1.042	1.023	1.027
±SE	0.018	0.029	0.032	0.028	0.033	0.025	0.029	0.037
SBI-HD	0.976	0.939	0.943	0.949	0.905	0.939	0.93	0.929
±SE	0.071	0.07	0.078	0.095	0.079	0.104	0.099	0.09
***P* val. Sham vs SBI-HD**	1	1	1	1	0.831	0.82	0.542	0.92
***P* val. Sham vs SBI-LD**	1	1	1	1	1	1	1	1
***P* val. SBI-LD vs SBI-HD**	1	1	1	1	0.672	0.251	0.471	0.898
**Theta power within 24 hours**								
Sham	0.959	0.957	0.96	0.966	0.971	0.973	0.973	0.968
±SE	0.021	0.016	0.014	0.013	0.009	0.011	0.01	0.011
SBI-LD	1.012	1.012	1.034	1.025	1.023	1.014	1.009	1.021
±SE	0.016	0.017	0.01	0.011	0.013	0.012	0.011	0.01
SBI-HD	1.006	1.012	0.965	0.968	0.986	0.965	0.953	0.957
±SE	0.06	0.062	0.078	0.082	0.076	0.082	0.08	0.084
***P* val. Sham vs SBI-HD**	0.953	0.259	0.934	1	0.179	0.979	0.955	0.836
***P* val. Sham vs SBI-LD**	0.356	0.225	**0.025**	**0.057**	**0.043**	0.158	0.282	**0.056**
***P* val. SBI-LD vs SBI-HD**	1	1	0.612	0.62	1	1	1	0.943
**Alpha power within 24 hours**								
Sham	0.987	0.983	0.994	0.99	0.979	0.97	0.965	0.957
±SE	0.03	0.02	0.017	0.019	0.018	0.023	0.023	0.019
SBI-LD	0.947	0.947	0.969	0.953	0.949	0.94	0.934	0.93
±SE	0.023	0.025	0.02	0.025	0.02	0.022	0.02	0.025
SBI-HD	0.879	0.869	0.858	0.857	0.867	0.867	0.891	0.878
±SE	0.022	0.025	0.024	0.029	0.035	0.029	0.027	0.03
***P* val. Sham vs SBI-HD**	0.143	**0.023**	**0.007**	**0.012**	**0.018**	0.064	0.186	0.227
***P* val. Sham vs SBI-LD**	0.613	0.92	1	0.737	1	1	1	1
***P* val. SBI-LD vs SBI-HD**	1	0.304	0.063	0.264	0.209	0.529	0.958	1
**Beta power within 24 hours**								
Sham	1.025	1.045	1.055	1.068	1.074	1.086	1.083	1.085
±SE	0.057	0.045	0.051	0.047	0.049	0.053	0.05	0.047
SBI-LD	1.004	1	0.968	0.967	0.985	0.982	0.998	0.975
±SE	0.025	0.029	0.029	0.03	0.03	0.026	0.026	0.032
SBI-HD	1.137	1.163	1.229	1.207	1.181	1.199	1.209	1.224
±SE	0.056	0.049	0.064	0.06	0.066	0.063	0.068	0.076
***P* val. Sham vs SBI-HD**	0.567	0.409	0.431	0.617	0.55	0.507	0.485	0.394

***P* val. Sham vs SBI-LD**	1	1	0.437	0.22	0.465	0.434	0.488	0.35
***P* val. SBI-LD vs SBI-HD**	0.245	0.119	**0.029**	**0.021**	**0.04**	**0.033**	**0.036**	**0.017**
**Gamma power within 24 hours**								
Sham	0.996	1.005	1.009	0.993	1	1.014	0.994	1.005
±SE	0.049	0.034	0.039	0.031	0.03	0.028	0.028	0.029
SBI-LD	0.985	0.982	0.984	0.973	0.944	0.977	0.986	0.977
±SE	0.021	0.013	0.021	0.015	0.016	0.008	0.01	0.023
SBI-HD	0.962	0.976	0.93	0.905	0.973	0.939	0.932	0.937
±SE	0.057	0.047	0.094	0.093	0.075	0.106	0.098	0.091
***P* val. Sham vs SBI-HD**	0.946	1	1	0.634	1	1	1	1
***P* val. Sham vs SBI-LD**	0.671	0.868	0.897	0.641	0.354	0.693	1	1
***P* val. SBI-LD vs SBI-HD**	1	1	1	1	0.805	1	1	1
